# Supplementary material for: circDCUN1D4 suppresses tumor metastasis and glycolysis in lung adenocarcinoma by stabilizing TXNIP expression
Source: Mol Ther Nucleic Acids. 2020 Nov 17;23:355–68. doi: 10.1016/j.omtn.2020.11.012 (PMC7779544; doi:10.1016/j.omtn.2020.11.012)
Supplement: Document S1. Supplemental materials and methods, Figures S1–S4, and Tables S1–S6 [file mmc1.pdf]

**OMTN, Volume *23***

## Supplemental Information

# circDCUN1D4 suppresses tumor metastasis and glycolysis in lung adenocarcinoma by stabilizing TXNIP expression

**Yingkuan Liang, Hui Wang, Bing Chen, Qixing Mao, Wenjie Xia, Te Zhang, Xuming Song, Zeyu Zhang, Lin Xu, Gaochao Dong, and Feng Jiang**

# Supplementary Materials and Methods

**Actinomycin D and RNase R treatment**

Cells were planted into six-well plates. Up to 60% confluency after 24 h, cells were treated with 5μg/ml Actinomycin D at indicated time points.

Total RNA (2μg) was incubated with 3 U/μg of RNase R (Sigma) for 15 min at 37 °C. After treatment with Actinomycin D or RNase R, the RNA expression levels of circDCUN1D4 and other mRNAs were analyzed by qRT-PCR.

**Transwell migration and invasion assay**

$4 \times 10^4$ cells, suspended in medium without FBS, were seeded into transwell chambers (Corning), with or without Matrigel (Sigma-Aldrich) coating. The lower chamber contained medium with 10% FBS as chemokine. Twenty-four hours later, the migratory or invasive cells on the lower surface of the chamber were photographed and counted in 10 random microscopic fields after crystal violet staining.

**Wound-healing assay**

The transfected cells were cultured in 6-well plates. After the cells reached 90% confluence, a standard 200μl pipette tip was subsequently utilized to scratch linear wounds. In addition, the cell monolayers were cultivated in FBS-free medium. After scratching, the images of the wound closure were captured at 0, and 24h.

**Real-time cell analysis**

The CIM-plate16 contains 16 wells, each a modified Boyden chamber, which can be used independently but simultaneously to measure cell migration in real-time through 8μm pores of a polyethylene terephthalate membrane on to gold electrodes on the

underside of the membrane using the xCELLigence system (ACEA Biosciences, USA). Experiments were set up according to the manufacturer's instructions with the membrane uncoated (migration) or coated with growth-factor-reduced-matrigel (invasion) (BD BioSciences, UK) (20 μl 1:40 diluted matrigel per well on the upper surface). Cell index (electrical impedance) was monitored every 30 min for the duration of the experiment. Traces show the average of quadruplicate wells.

**RNA-Fluorescence in situ hybridization assay and Fluorescence immunocytochemical staining**

RNA-Fluorescence in situ hybridization (FISH) assays were performed using a RNA-FISH kit (GenePharma, China) according to the manufacturer's instructions. Cy3-labeled antisense probe was synthesized by GenePharma company (Suzhou, China) against circDCUN1D4. The sequence is listed in Table S2. In briefly, A549 cells were fixed with 4% paraformaldehyde. After pre-hybridization with 1× PBS/0.5% Triton X-100, cells were blocked and hybridized in hybridization buffer with Cy3-labeled antisense probe at 37 °C overnight. Then cells were incubated with specific antibodies for HuR (ab200342, 1:200 dilution) at 4 °C overnight. Cells were treated with tyramide-conjugated goat anti-rabbit IgG (Invitrogen 1:10000 dilution) and DAPI (300 nmol/L) staining. The images were photographed under a Nikon A1Si Laser Scanning Confocal Microscope (Nikon Instruments Inc, Japan).

**Luciferase reporter assay**

Human HuR luciferase reporter was constructed according to the previous article[2]. The TXNIP binding sites of circDCUN1D4 were analyzed by StarBase

(http://starbase.sysu.edu.cn/index.php). The different fragment sequences were synthesized and then inserted into the psiCHECK-2 vector (Promega). All vectors were verified by sequencing and luciferase activity was assessed using the Dual Luciferase Assay Kit (Promega) according to the manufacturer's instructions. The sequences were provided in Table S2

**Microarray assay**

The Agilent human mRNA array was designed with 8 identical arrays per slide (8 × 60 K format), with each array containing probes interrogating approximately 27958 Entrez Gene RNAs and 7419 long intergenic noncoding RNA. The array also contains 1280 Agilent control probes. Total RNA containing small RNA was extracted from cells by using TRIzol reagent (Invitrogen) according to the manufacturer's protocol. The purity and concentration of RNA were determined from optical density 260/280 readings using a spectrophotometer (NanoDrop ND-1000). RNA integrity was determined by 1% formaldehyde denaturing gel electrophoresis.

**Tissue microarray and in situ hybridization assay**

Tissue microarray (TMA) was obtained from Outdo Biotech Co. Ltd. (Shanghai, China). Ninety-two pairs of LUAC tissues and their paired peripheral normal lung tissues were used to construct the TMA. Special biotin labelled probe against circDCUN1D4 was synthesized by GenePharma company (Suzhou, China). The sequence is provided in Table S2. After incubating with the biotin labelled probe, the TMA was then stained with DAB and hematoxylin, dehydrated and covered. The TMA staining scores were evaluated by two independent observers blinded to the clinicopathological data. The

staining scores were based on two indicators: the proportion of positively stained cells and the staining intensity. The proportion of positively stained cells was evaluated with five scoring levels: 0, <10%; 1, 10–25%; 2, 25–50%; 3, 50–75%; and 4, >75%. The staining intensity was scored with the following point system: 0 (no staining), 1 (yellow), 2 (yellow-brown) and 3 (dark brown). The products of the above two indicators were considered the total score.

**Files uploaded separately in Excel Format:**

**Additional Dataset 1**. Fold enrichment of 152 circRNA in HuR CLIP.

**Additional Dataset 2.** 661 genes altered by circDCUN1D4.

# Supplementary Figures

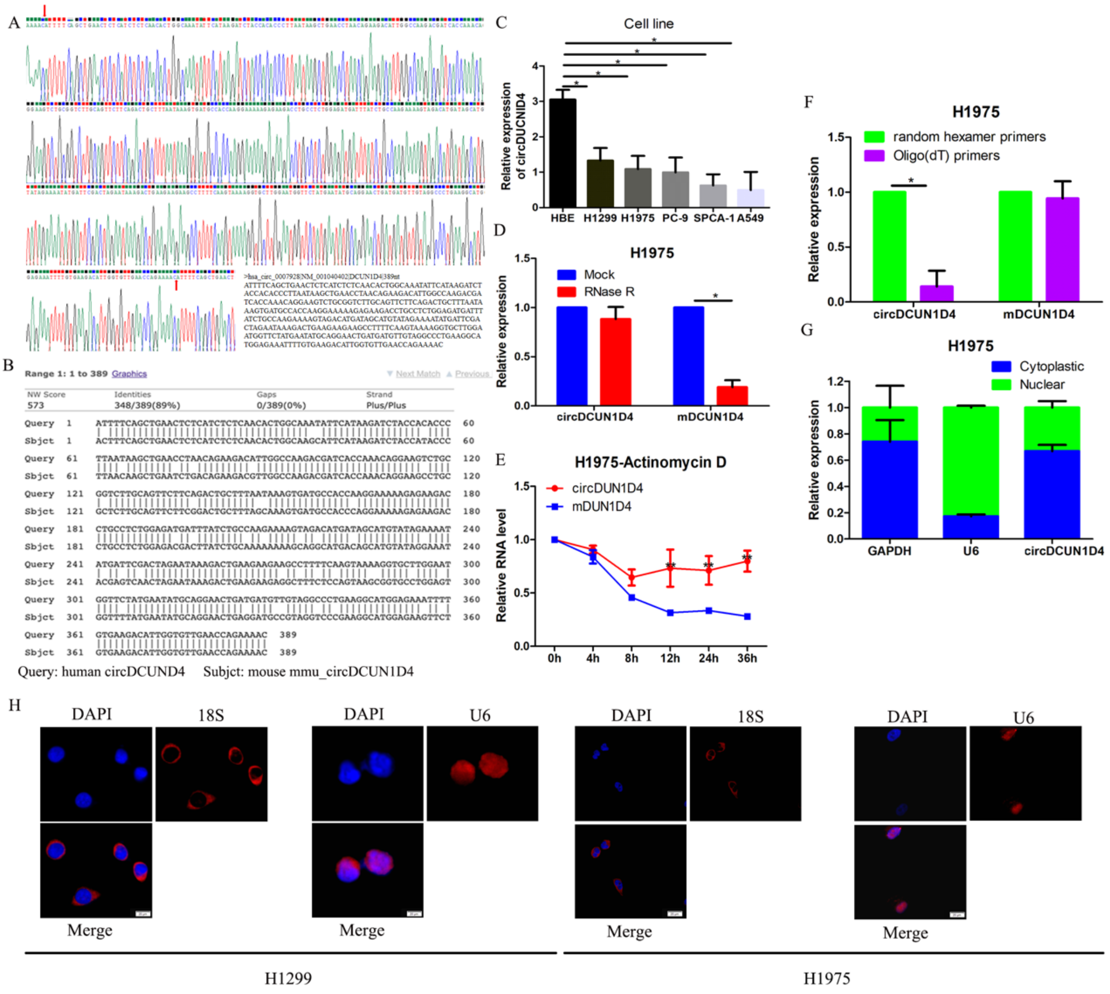


**Fig. S1 Characterization of circDCUN1D4. A** The full length of circDCUN1D4. **B** Conservative analysis of circDCUN1D4 between humans and mice. **C** qRT-PCR assay detected the expression of circDCUN1D4 in a variety of LUAD cell lines and a normal lung cell line HBE. (mean ± SD, n = 4) **D** qRT-PCR for the abundance of circDCUN1D4 and DCUN1D4 in H1975 cells treated with RNase R compared with the mock group. (mean ± SD, n = 4) **E** qRT-PCR for the abundance of circDCUN1D4 and DCUN1D4 in H975 cells treated with Actinomycin D at the indicated time point. (mean ± SD, n = 4) **F** Random hexamer or oligo (dT)18 primers were used in the reverse transcription experiment. The relative RNA levels were analyzed by qRT-PCR and normalized to the value using random hexamer primers. (mean ± SD, n = 4) **G** qRT-PCR for the distribution of circDCUN1D4, GAPDH, and U6 in the cytoplasmic and nuclear fractions of H1975 cells. **H** The 18S and U6 were applied as positive control for

cytoplasm and nucleus respectively in RNA-FISH assay. Student's t test and analysis of variance compared the difference in C, D, and E. *P < 0.05 vs. HBE, mock, mDCUN1D4.

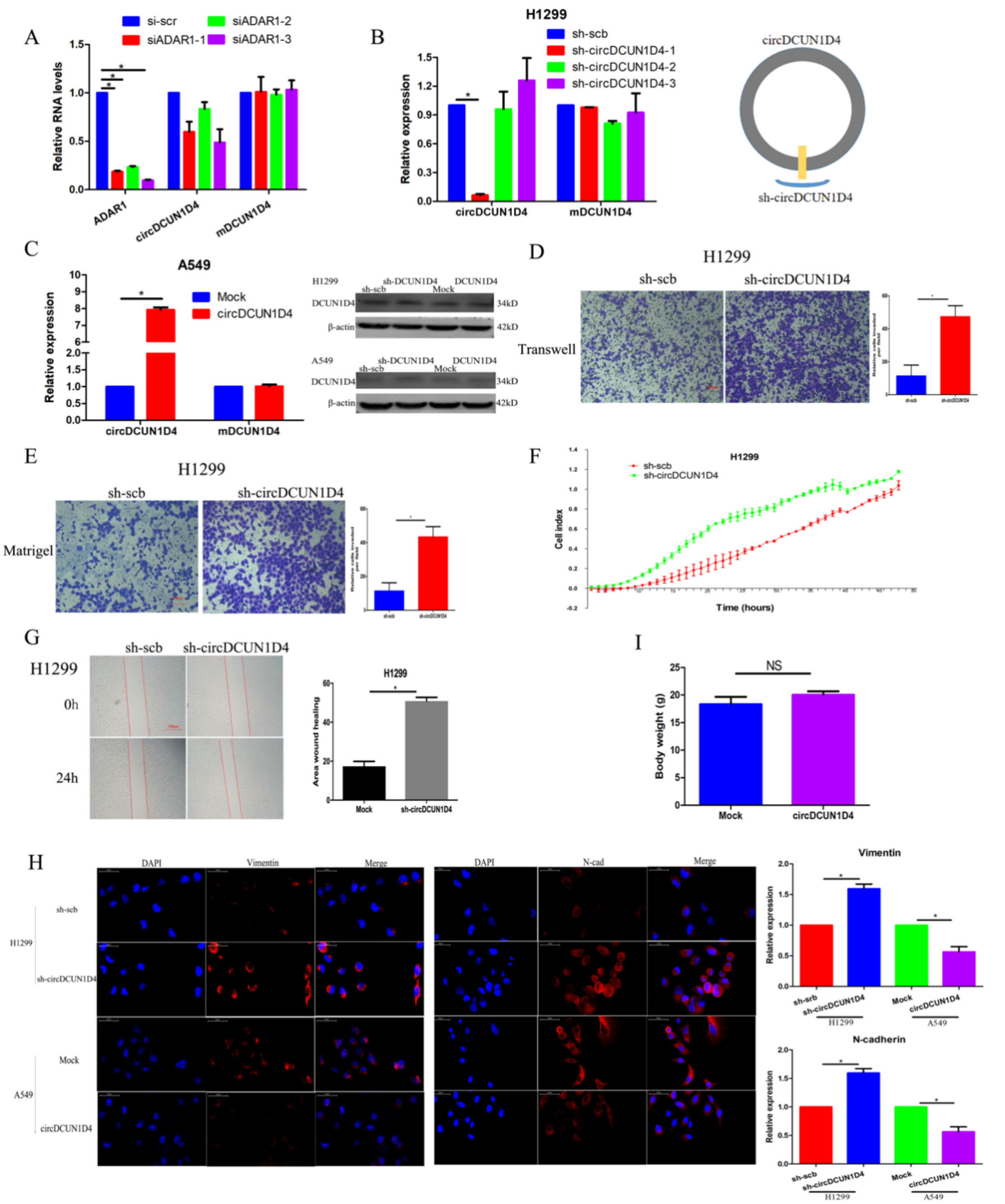


**Fig. S2 Effects of circDCUN1D4 on the invasion of cancer cells. A** qRT-PCR assay showing the transcript levels of EIF4A3, QKI, and ADAR1 in cancer cells stably transfected with si-scb, or siEIF4A3, or siQKI, or siADAR1, respectively. (mean ± SD, n = 4) **B** qRT-PCR and western blot assays showing the transcript and protein levels of DCUNID4 in cancer cells stably transfected with mock, circDCUN1D4, sh-Scb, or sh-DCUN1D4. (mean ± SD, n = 4)**C-F** Representative images and quantification of transwell (C), matrigel (D), C-plate (E) and wounding healing (F) assays showing the invasion of H1299 cells stably transfected with sh-scb or sh-DCU1ND4 (mean ± SD,

n=4). Scale bar: 100 μm. **G** Representative images (left panel) and quantification (right panel) of immunofluorescence staining assay showing the expression of Vimentin or N-cadherin of A549 and H1299 cells stably transfected with mock, circDCUN1D4, scramble shRNA (sh-scb), or sh-circDCUN1D4 (mean ± SD, n=4). **H** The body weight of mice after tail vein injection of A549 cells stably transfected with mock and circDCUN1D4 (n=4 for each group). Student's t test and analysis of variance compared the difference in A-G. *P < 0.05 vs. si-scb, mock, sh-scb.

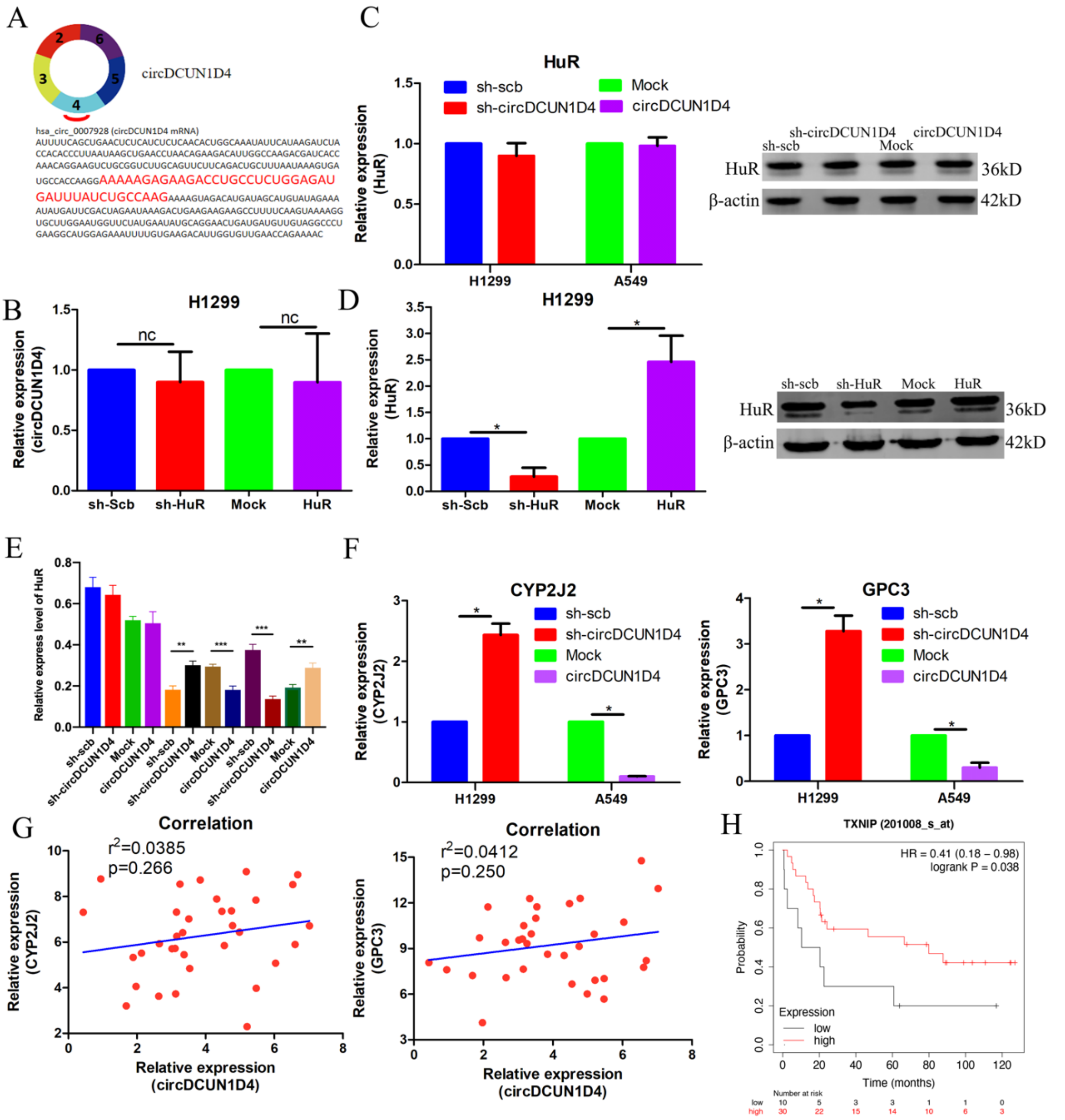


**Fig. S3 Effects of circDCUN1D4 on the expression of HuR, CYP2J2 or GPC3 in cancer cells. A** The sequences of circDCUN1D4 interacts with HuR protein. **B** qRT-PCR assay showing the expression of circDCUN1D4 in cancer cells stably transfected with mock, HuR, sh-scb, or sh-HuR. (mean ± SD, n=4) **C** qRT-PCR and western blot assays showing the transcript and protein levels of HuR in cancer cells stably transfected with mock, circDCUN1D4, sh-Scb, or sh-DCUN1D4. (mean ± SD, n=4) **D** qRT-PCR and western blot assays showing the transcript and protein levels of HuR in cancer cells stably transfected with mock, HuR, sh-scb, or sh-HuR. (mean ± SD, n=4) **E** The quantification of Western blot indicating the expression of HuR in total lysates

or subcellular fractions of H1299 cells stably transfected with mock, circDCUN1D4, sh-scb, or sh-circDCUN1D4 vectors. **F** qRT-PCR detection of CYP2J2 or GPC3 in cancer cells stably transfected with mock, circDCUN1D4, scramble shRNA (sh-scb), or sh-circDCUN1D4 (mean ± SD, n=4). **G** The correlation between the relative expression of CYP2J2 or GPC3 and circDCUN1D4 in 34 LUAD tissues. **H** Kaplan-Meier curves indicating the overall (OS) survival of TXNIP in lung cancer cases derived from Kaplam-Meier Plotter. Student's t test and analysis of variance compared the difference in A-F. ***P<0.001, **P<0.01, *P < 0.05 vs. mock, sh-scb.

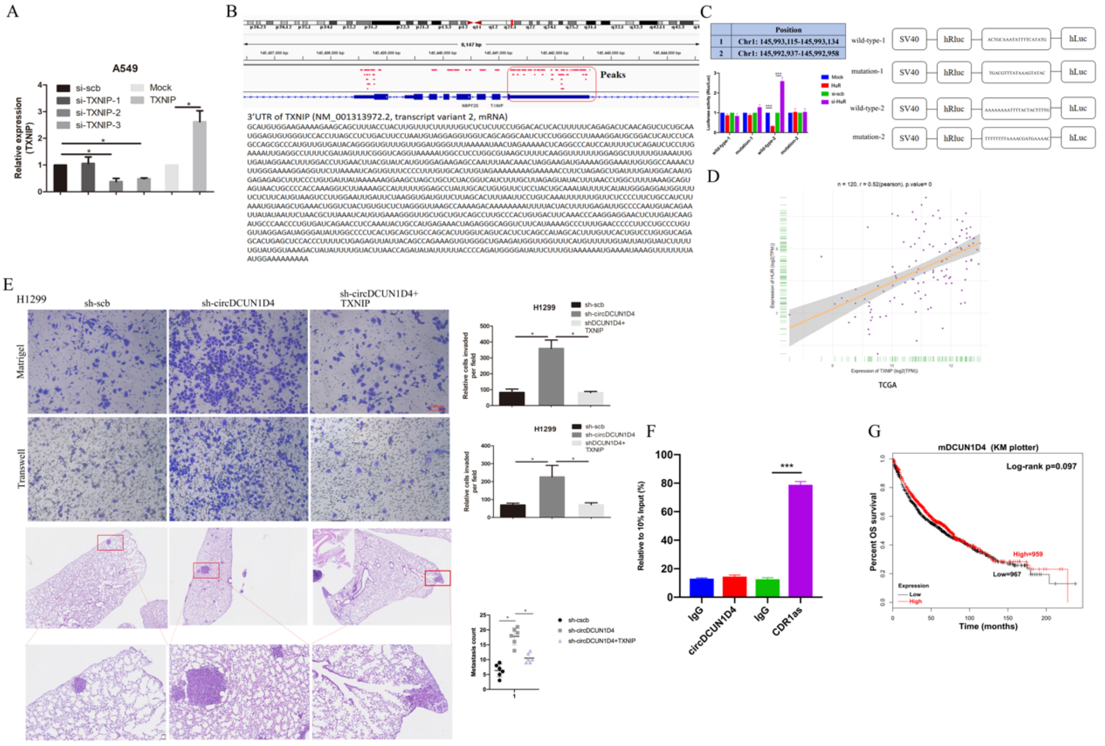


**Fig. S4 circDCUN1D4 suppresses metastasis depend on TXNIP pathway. A** qRT-PCR assay showing the expression of TXNIP in cancer cells stably transfected with Mock, TXNIP, si-scb, si-TXNIP-1, si-TXNIP-2, and si-TXNIP-3. (mean ± SD, n=4). **B** IGV showing the peaks localized in 3'UTP of TXNIP which interacts with HuR protein. **C** Dual-luciferase assay indicating the interacted region between 3'UTR region of TXNIP and HuR in H1299 cells stably transfected with mock, HuR, si-scb, or si-HuR (mean ± SD, n=4). **D** The expression level of TXNIP was significantly positive relative to the expression level of HuR. **E** Representative images (left panel) and quantification (right panel) of transwell, matrigel, and *in vivo* assays showing the invasion of H1299 cells stably transfected with sh-scb, sh-circDCUN1D4 or sh-circDCUN1D4 plus TXNIP (mean ± SD, n=4). Scale bar: 100 μm. **F** RIP assay showing that the RNA binding protein Ago2 could not interacts with circDCUN1D4. **G** Kaplan-Meier curves indicating the overall (OS) survival of DCUN1D4 in lung cancer cases derived from Kaplam-Meier Plotter. Student's t test and analysis of variance compared the difference in A, E and F. ***P<0.001, *P < 0.05 vs. mock, sh-scb, sh-circDCUN1D4, AND IgG. Log-rank test for survival comparison in G.

# Supplementary Tables

**Supplementary Table S1 Primer sets used for RT-PCR, qRT-PCR, and RIP**

| **Primer set** | **Primers** | **Sequence** | **Application** |
|---|---|---|---|
| hsa_circ_0001212 | Forward | 5' -GTGAAGCGAGACAGTGGTGA-3' | qRT-PCR |
| | Reverse | 5' -ACCACAACCAGCTGACAAAA-3' | |
| hsa_circ_0002343 | Forward | 5' -GTGGTGTGCAGGTGAACAAG-3' | qRT-PCR |
| | Reverse | 5' -ACAACACGCCAACTACCACA-3' | |
| hsa_circ_0007386 | Forward | 5' -CCCGGACAGCTATGAAACTC-3' | qRT-PCR |
| | Reverse | 5' -AGGAGCATAACCCTCGATCA-3' | |
| hsa_circ_0007928 (circDCUN1D4) | Forward | 5'- GTTGAACCAGAAAACATTTTC-3 | qRT-PCR, RIP RT-PCR |
| | Reverse | 5'- AGACTTCCTGTTTGGTGATC -3' | |
| hsa_circ_0012265 | Forward | 5'-GGTTCCTAAGCCTGTACCACC -3' | qRT-PCR |
| | Reverse | 5'-GCTGGTGCCAAGAATCTCCT-3' | |
| mDCUN1D4 | Forward | 5'-TGCAGGCAGTGGACAAGAAA-3' | qRT-PCR RT-PCR |
| | Reverse | 5'-GGATCTTGCTGACCACGACA-3' | |
| HuR | Forward | 5'-ATGTTCTCTCGGTTTGGGCG-3' | qRT-PCR |
| | Reverse | 5'-GTTCTGGTTGGGGTTGGCTG-3' | |
| 18S | Forward | 5' -CGAACGTCTGCCCTATCAACTT-3' | qRT-PCR |
| | Reverse | 5' -ACCCGTGGTCACCATGGTA-3' | |
| U6 | Forward | 5' -CTCGCTTCGGCAGCACA-3' | qRT-PCR |
| | Reverse | 5' -AACGCTTCACGAATTTGCG-3' | |
| GAPDH | Forward | 5' -AGAAGGCTGGGGCTCATTTG-3' | qRT-PCR RT-PCR |
| | Reverse | 5' -AGGGGCCATCCACAGTCTTC-3' | |
| DHX9 | Forward | 5' -TGCCTCCAAGAAAGTCCA-3' | qRT-PCR |
| | Reverse | 5' -TCCGCTTCCATTGTCGTAT-3' | |
| EIF4A3 | Forward | 5' -GGGGCATCTACGCTTACGG-3' | qRT-PCR |
| | Reverse | 5' -GCGATGACATCTCTCCCTTTGA-3' | |
| QKI | Forward | 5' -CAAACGGAACTCCTCACCC-3' | qRT-PCR |
| | Reverse | 5' -GCCACCGCACCTAATACAC-3' | |
| ADAR1 | Forward | 5' -CGAGAA TCCCAAACAAGGAA-3' | qRT-PCR |
| | Reverse | 5' -CTGGATTCCACAGGGATTGT-3' | |
| TXNIP | Forward | 5' -TGTGTGAAGTTACTCGTGTCAAA-3' | qRT-PCR |
| | Reverse | 5' -GCAGGTACTCCGAAGTCTGT-3' | |
| CYP2J2 | Forward | 5' -TCCATCCTCGGACTCTCCTAC-3' | qRT-PCR |
| | Reverse | 5' -GTCACCAAGCTCCAAGCTAAAA-3' | |
| GPC3 | Forward | 5' -CAGTAAGGACTGTGGCCGAAT-3' | qRT-PCR |
| | Reverse | 5' -AGCAGTACGTTCTCCATGTCAT-3' | |
| pre-TXNIP | Forward | 5' -AGAAGTTGACTTAGACGGATTGCT-3' | qRT-PCR |

| | | | |
|---|---|---|---|
| | Reverse | 5’ -ACAGACTATTTCAGTTCAGGTAAGA-3’ | |
| TXNIP-1 | Forward | 5’ -ACGCTTCTTCTGGAAGACCA-3’ | qRT-PCR |
| | Reverse | 5’ -AGGGGTATTGACATCCACCA-3’ | |
| TXNIP-2 | Forward | 5’ -GCAAGCCTAATGGCTACTCG-3’ | qRT-PCR |
| | Reverse | 5’ -AGGGGTATTGACATCCACCA-3’ | |
| 3’ UTR (TXNIP) | Forward | 5’ -GAGTGTGGGTCCACCTTAGC-3’ | qRT-PCR |
| | Reverse | 5’ -AGGGGTATTGACATCCACCA-3’ | |
| BEX2 | Forward | 5’ -AAAGAGGAACGAGCGTTAAACA(-3’ | qRT-PCR |
| | Reverse | 5’ -TCACTAACATTCAAAGGTAGGGC-3’ | |
| DDX6 | Forward | 5’ -ATGGGTCTGTCCAGTCAAAATG-3’ | qRT-PCR |
| | Reverse | 5’ -GGTGGTCATACTCTGTGCTTG-3’ | |
| FBXW2 | Forward | 5’ -TTCTTTCTCTGACGGACTTGC-3’ | qRT-PCR |
| | Reverse | 5’ -GCTTGAGGAGAGTCTCTAGGTT-3’ | |
| FXR1 | Forward | 5’ -GAGAGAAGATTTAATGGGCCTGG-3’ | qRT-PCR |
| | Reverse | 5’ -GCTCAATGGCGGTAACTCCA-3’ | |
| HK2 | Forward | 5’ -GAGCCACCACTCACCCTACT-3’ | qRT-PCR |
| | Reverse | 5’ -CCAGGCATTCGGCAATGTG-3’ | |
| IFI6 | Forward | 5’ -GGTCTGCGATCCTGAATGGG-3’ | qRT-PCR |
| | Reverse | 5’ -TCACTATCGAGATACTTGTGGGT-3’ | |
| LOX | Forward | 5’ -CGGCGGAGGAAAACTGTCT-3’ | qRT-PCR |
| | Reverse | 5’ -TCGGCTGGGTAAGAAATCTGA-3’ | |
| MGAT5 | Forward | 5’ -CTTCACTCCGTGGAAGTTGTC-3’ | qRT-PCR |
| | Reverse | 5’ -TGGATGGTAAAGTGCAGAAGC-3’ | |
| PCDH9 | Forward | 5’ -CTGCTCTGATTGCCTGTTTAAGG-3’ | qRT-PCR |
| | Reverse | 5’ -ACCAGTCTGTAGACAAGGCTG-3’ | |
| PLIN2 | Forward | 5’ -ATGGCATCCGTTGCAGTTGAT-3’ | qRT-PCR |
| | Reverse | 5’ -GGACATGAGGTCATACGTGGAG-3’ | |
| PML | Forward | 5’ -CGCCCTGGATAACGTCTTTTT-3’ | qRT-PCR |
| | Reverse | 5’ -CTCGCACTCAAAGCACCAGA-3’ | |
| SMAD2 | Forward | 5’ -CGTCCATCTTGCCATTCACG-3’ | qRT-PCR |
| | Reverse | 5’ -CTCAAGCTCATCTAATCGTCCTG-3’ | |
| STXBP4 | Forward | 5’ -CCTTGGCCTGAAGGTACTAGG-3’ | qRT-PCR |
| | Reverse | 5’ -AGCAGATTCTAACCTCAACTTGG-3’ | |
| ZNF680 | Forward | 5’ -TGGGTGCTCCAGCCTTACT-3’ | qRT-PCR |
| | Reverse | 5’ -GCAGGCCAGTTAAAAACATTGC-3’ | |
| UQCRB | Forward | 5’ -GGTAAGCAGGCCGTTTCAG-3’ | qRT-PCR |
| | Reverse | 5’ -AGGTCCAGTGCCCTCTTAATG-3’ | |
| SMC2 | Forward | 5’ -ACAACACCAGAGTACAGGATCT-3’ | qRT-PCR |
| | Reverse | 5’ -CGGCCCTGCATGATGAGAA-3’ | |
| DCUN1D4-intron2 | Forward | 5’ -CAGATGCAGGGTGTACAAGG-3’ | qRT-PCR |
| | Reverse | 5’ -TCAGTAAACAGAGGGGCAACT-3’ | |

| | | | |
|---|---|---|---|
| DCUN1D4-intron6 | Forward | 5’ - CTTCGTGCCCCTCAGTAGTT-3’ | qRT-PCR |
| | Reverse | 5’ - CATACCATTGCACTCCAGCC-3’ | |

**Supplementary Table S2 Oligonucleotide sets used for constructs, guide DNA, and probe**

| **Oligo Set** | **Sequences** |
|---|---|
| pcDNA3.1(+)-circDCUN1D4 (positive) | 5’-tactaatgactttttttttatacttcagATTTTCAGCTGAACTCTCAT-3’(sense) |
| | 5’-gcctaattcttttccttgcttcttacGTTTTCTGGTTCAACACCAA-3’ (antisense) |
| sh-circDCUN1D4-1 | 5'-CACCGCAGAAAACATTTTCAGCTGAATTCAAGAGATTCAGCTGAAAATGTTTTCTGTTTTTTG-3' (sense) |
| | 5'-GATCCAAAAAACAGAAAACATTTTCAGCTGAATCTCTTGAATTCAGCTGAAAATGTTTTCTGC-3' (antisense) |
| sh-circDCUN1D4-2 | 5'-CACCGCAAACATTTTCAGCTGAACTCAAGAGAAGUUCAGCUGAAAAUGTTTTCTTTTTTG-3' (sense) |
| | 5'-GATCCAAAAAACAAACATTTTCAGCTGAACTCTCTTGAAAGTTCAGCTGAAAATGTTTTC-3' (antisense) |
| sh-circDCUN1D4-3 | 5'-CACCGCCCAGAAAACATTTTCAGCTTCAAGAGAAGCTGAAAATGTTTTCTGGTTTTTTG-3' (sense) |
| | 5'-GATCCAAAAAACCCAGAAAACATTTTCAGCTCTCTTGAAAGCUGAAAATGTTTCTGGC-3' (antisense) |
| sh-scb | 5'-CACCGCTTCTCCGAACGTGTCACGTTTCAAGAGAACGTGACACGTTCGGAGAATTTTTTG-3' (sense) |
| | 5'-GATCCAAAAAACTTCTCCGAACGTGTCACGTTTCAAGAGAACGTGACACGTTCGGAGAAC-3' (antisense) |
| sh-HuR | 5’-CACCGCCCATCACAGTGAAGTTTGCATTCAAGAGATGCAAACTTCACTGTGATGGGTTTTTTG-3’ (sense) |
| | 5’-GATCCAAAAAACCCATCACAGTGAAGTTTGCATCTCTTGAATGCAAACTTCACTGTGATGGGC-3’ (antisense); |
| psiCHECK2-HuR-Rluc | 5'-TCGAGTGGATACCGTTCGTTGTTAACCGTTGATAGCCTTGGTTTTTAAGCCGTATATGGCTGTAAATAATTGC-3' (sense) |
| | 5'-GGCCGCAATTATTTACAGCCATATACGGCTTAAAAACCAAGGCTATCAACGGTTAACAACGAACGGTATCCAC-3' (antisense) |
| pcDNA3.1(+)-Flag-HuR | 5'-CCGGAATTCATGTCTAATGGTTATGAAGACC-3' (sense); |
| | 5'-CCGCTCGAGTTATTTGTGGGACTTGTTGGTT-3' (antisense) |

| | |
|---|---|
| pcDNA3.1(+)-Flag-HuR(ΔRRM3) | 5'-CCGGAATTCATGTCTAATGGTTATGAAGACC-3' (sense); |
| | 5'-CCGCTCGAGCCAGCCGGAGGAGGCGTTTCCT-3' (antisense) |
| pcDNA3.1(+)-Flag- HuR (RRM2 + Hinge) | 5'-CCGGAATTCATCAAAGACGCCAACTTGTACA-3' (sense); |
| | 5'-CCGCTCGAGCCAGCCGGAGGAGGCGTTTCCT-3' (antisense) |
| pcDNA3.1(+)-Flag- HuR (Hinge) | 5'-CCGGAATTCAACAAAAACGTGGCACTCCTCT-3' (sense); |
| | 5'-CCGCTCGAGCCAGCCGGAGGAGGCGTTTCCT-3' (antisense) |
| psiCHECK2-TXNIP-WT-Rluc | GCATGTGGAAGAAAAGAAGCAGCTTTACCTACTTGTTTCTTTTTGTCTCTCTTC<br>CTGGACACTCACTTTTTCAGAGACTCAACAGTCTCTGCAATGGAGTGTGGGTCC<br>ACCTTAGCCTCTGACTTCCTAATGTAGGAGGTGGTCAGCAGGCAATCTCCTGGG<br>CCTTAAAGGATGCGGACTCATCCTCAGCCAGCGCCCATGTTGTGATACAGGGGT<br>GTTTGTTGGATGGGTTTAAAAATAACTAGAAAAACTCAGGCCCATCCATTTTCT<br>CAGATCTCCTTGAAAATTGAGGCCTTTTCGATAGTTTCGGGTCAGGTAAAAATG<br>GCCTCCTGGCGTAAGCTTTTCAAGGTTTTTTGGAGGCTTTTTGTAAATTGTGAT<br>AGGAACTTTGGACCTTGAACTTACGTATCATGTGGAGAAGAGCCAATTTAACAA<br>ACTAGGAAGATGAAAAGGGAAATTGTGGCCAAAACTTTGGGAAAAGGAGGTTCT<br>TAAAATCAGTGTTTCCCCTTTGTGCACTTGTAGAAAAAAAAGAAAAACCTTCTA<br>GAGCTGATTTGATGGACAATGGAGAGAGCTTTCCCTGTGATTATAAAAAAGGAA<br>GCTAGCTGCTCTACGGTCATCTTTGCTTAGAGTATACTTTAACCTGGCTTTTAA<br>AGCAGTAGTAACTGCCCCACCAAAGGTCTTAAAAGCCATTTTTGGAGCCTATTG<br>CACTGTGTTCTCCTACTGCAAATATTTTCATATGGGAGGATGGTTTTCTCTTCA<br>TGTAAGTCCTTGGAATTGATTCTAAGGTGATGTTCTTAGCACTTTAATTCCTGT<br>CAAATTTTTTGTTCTCCCCTTCTGCCATCTTAAATGTAAGCTGAAACTGGTCTA<br>CTGTGTCTCTAGGGTTAAGCCAAAAGACAAAAAAAATTTTACTACTTTTGAGAT<br>TGCCCCAATGTACAGAATTATATAATTCTAACGCTTAAATCATGTGAAAGGGTT<br>GCTGCTGTCAGCCTTGCCCACTGTGACTTCAAACCCAAGGAGGAACTCTTGATC<br>AAGATGCCCAACCCTGTGATCAGAACCTCCAAATACTGCCATGAGAAACTAGAG<br>GGCAGGTCTTCATAAAAGCCCTTTGAACCCCCTTCCTGCCCTGTGTTAGGAGAT<br>AGGGATATTGGCCCCTCACTGCAGCTGCCAGCACTTGGTCAGTCACTCTCAGCC<br>ATAGCACTTTGTTCACTGTCCTGTGTCAGAGCACTGAGCTCCACCCTTTTCTGA<br>GAGTTATTACAGCCAGAAAGTGTGGGCTGAAGATGGTTGGTTTCATGTTTTTGT<br>ATTATGTATCTTTTTGTATGGTAAAGACTATATTTTGTACTTAACCAGATATAT<br>TTTTACCCCAGATGGGGATATTCTTTGTAAAAAATGAAAATAAAGTTTTTTTAA<br>TGGAAAAAAAAA |

| | |
|---|---|
| psiCHECK2-TXNIP-Mut-Rluc | GCATGTGGAAGAAAAGAAGCAGCTTTACCTACTTGTTTCTTTTTGTCTCTCTTC<br>CTGGACACTCACTTTTTCAGAGACTCAACAGTCTCTGCAATGGAGTGTGGGTCC<br>ACCTTAGCCTCTGACTTCCTAATGTAGGAGGTGGTCAGCAGGCAATCTCCTGGG<br>CCTTAAAGGATGCGGACTCATCCTCAGCCAGCGCCCATGTTGTGATACAGGGGT<br>GTTTGTTGGATGGGTTTAAAAATAACTAGAAAAACTCAGGCCCATCCATTTTCT<br>CAGATCTCCTTGAAAATTGAGGCCTTTTCGATAGTTTCGGGTCAGGTAAAAATG<br>GCCTCCTGGCGTAAGCTTTTCAAGGTTTTTTGGAGGCTTTTTGTAAATTGTGAT<br>AGGAACTTTGGACCTTGAACTTACGTATCATGTGGAGAAGAGCCAATTTAACAA<br>ACTAGGAAGATGAAAAGGGAAATTGTGGCCAAAACTTTGGGAAAAGGAGGTTCT<br>TAAAATCAGTGTTTCCCCTTTGTGCACTTGTAGAAAAAAAAGAAAAACCTTCTA<br>GAGCTGATTTGATGGACAATGGAGAGAGCTTTCCCTGTGATTATAAAAAAGGAA<br>GCTAGCTGCTCTACGGTCATCTTTGCTTAGAGTATACTTTAACCTGGCTTTTAA<br>AGCAGTAGTAACTGCCCCACCAAAGGTCTTAAAAGCCATTTTTGGAGCCTATTG<br>CACTGTGTTCTCCTACTGCAAATATTTTCATATGGGAGGATGGTTTTCTCTTCA<br>TGTAAGTCCTTGGAATTGATTCTAAGGTGATGTTCTTAGCACTTTAATTCCTGT<br>CAAATTTTTTGTTCTCCCCTTCTGCCATCTTAAATGTAAGCTGAAACTGGTCTA<br>CTGTGTCTCTAGGGTTAAGCCAAAAGACAAAAAAAATTTTACTACTTTTGAGAT<br>TGCCCCAATGTACAGAATTATATAATTCTAACGCTTAAATCATGTGAAAGGGTT<br>GCTGCTGTCAGCCTTGCCCACTGTGACTTCAAACCCAAGGAGGAACTCTTGATC<br>AAGATGCCCAACCCTGTGATCAGAACCTCCAAATACTGCCATGAGAAACTAGAG<br>AAACCTTTTCATAAAAGCCCTTTGAACCCCCTTCCTGCCCTGTGTTAGGAGAT<br>AGGGATATTGGCCCCTCACTGCAGCTGCCAGCACTTGGTCAGTCACTCTCAGCC<br>ATAGCACTTTGTTCACTGTCCTGTGTCAGAGCACTGAGCTCCACCCTTTTCTGA<br>GAGTTATTACAGCCAGAAAGTGTGGGCTGAAGATGGTTGGTTTCATGTTTTTGT<br>ATTATGTATCTTTTTGTATGGTAAAGACTATATTTTGTACTTAACCAGATATAT<br>TTTTACCCCAGATGGGGATATTCTTTGTAAAAAATGAAAATAAAGTTTTTTTAA<br>TGGAAAAAAAAA |
| psiCHECK2-AluJo-WT-Rluc | GCTTACATTTTTATTGCCTAATATATATGCAAACATACTTTACTTCAAGTTGAG<br>GCTGCGCGTAGTGGCCCACACCTTTAATCCCAACATTTTGGGAGGCTGAGGCAG<br>GAGGATCACTTGAACCCAGGAGTTCAAGACCAGCCTGGGAAACATAGAAAGACC<br>CTGTCTCTACAAGAAAAAAAAAAAGAAAAATTAGCTGTGCATGATGGTGCACAC |

| | |
|---|---|
| psiCHECK2-AluJo-Mut-Rluc | GCTTACATTTTTATTGCCTAATATATATGCAAACATACTTTACTTCAAGTTGAG<br>GCTGCGCGTAGTGGCCCACACCTTTAAAGGGTTGATTTTGGGAGGCTGAGGCAG<br>GAGGATCACTTGAACCCAGGAGTTCAAGACCAGCCTGGGAAACATAGAAAGACC<br>CTGTCTCTACAAGAAAAAAAAAAAGAAAAATTAGCTGTGCATGATGGTGCACAC |
| psiCHECK2-AluSc-WT-Rluc | GTAGCTGGGACTACAGGTGTGCGCCACCATGCTTGGCTAATGTTTTTTGTATTT<br>TAGTAGAGATGGGATTTCACCATGTTGGCCAGATGTCTCTATCTCCTGACCTTG<br>TGATCTGCCCGGCTCAGCCTCCCAAAGTGCTGGGATTATAGGCGTGAGCCACAG<br>CACCCGGCCATGATTGCTTTTTATAATGTAAAAGCCCTAGGTATTTAT |

| | |
|---|---|
| psiCHECK2-AluSc-WT-Rluc | GTAGCTGGGACTACAGGTGTGCGCCACCATGCTTGGCTAATGTTTTTTGTATTTTAGTAGAGATGGGATTTCACCATGTTGGCCAGATGTCTCTATCTCCTGACCTTGTGATCTGCCCGGCTCAGCCAGGGTTTGTGCTGGGATTATAGGCGTGAGCCACAGCACCCGGCCATGATTGCTTTTTATAATGTAAAAGCCCTAGGTATTTAT |
| CircDCUN1D4 junction probe (northern blot) | 5'-GTTGAGAGATGAGAGTTCAGCTGAAAATGTTTTCTGGTTCAACACCAATGTCTTC- DIG-3' |
| CircDCUN1D4 junction probe (pull down) | 5'-GTTGAGAGATGAGAGTTCAGCTGAAAATGTTTTCTGGTTCAACACCAATGTCTTC-3' |
| CircDCUN1D4 Scramble probe | 5'-GTGTAACACGTCTATACGCCCA-3' |
| CircDCUN1D4 FISH Probe | 5'-GAAAACAUUUUCAGCUGAATT-3' (sense) |
| | 5'-UUCAGCUGAAAAUGUUUUCUG-3' (antisense) |
| CircDCUN1D4 ISH Probe | 5'-GTTGAGAGATGAGAGTTCAGCTGAAAATGTTTTCTGGTTCAACACCAATGT CTTC-3' |

**Supplementary Table S3  Target sequences of siRNA**

| **Oligo Set** | **Target sequences** |
|---|---|
| si-ADAR1-1 | GCAGAGTCAGCATATATGA |
| si-ADAR1-2 | GGCCCGAGATATAAATGCT |
| si-ADAR1-3 | GGCTCTCCGTGTCTTGATT |
| si-DHX9-1 | CGAACACCATTGCATGAAA |
| si-DHX9-2 | GGACTAGTAGCAACATTGA |
| si-DHX9-3 | GCATGGACCTCAAGAATGA |
| si-QKI-1 | GAAGCTGGTTTAATCTATA |
| si-QKI-2 | GACCTATTGTTCAGTTACA |
| si-QKI-3 | GAAACCGGATGTAAAATCA |
| si-EIF4A3-1 | CGAGCAATCAAGCAGATCA |
| si-EIF4A3-2 | GCTGGATTACGGACAGCAT |
| si-EIF4A3-3 | CTCTCGGTGACTACATGAA |
| si-TXNIP-1 | CAACATCCTTCGAGTTGAA |
| si-TXNIP-2 | ACACGCTTCTTCTGGAAGA |
| si-TXNIP-3 | GTCTGTCTCTGCTCGAATT |

**Supplementary Table S4 candidate targets of circDCUN1D4**

| | | |
|---|---|---|
| BEX2 | CYP2J2 | DDX6 |
| FBXW2 | FXR1 | GPC3 |
| HK2 | IFI6 | LOX |
| MGAT5 | PCDH9 | PLIN2 |
| PML | SMAD2 | SMC2 |
| STXBP4 | TXNIP | UQCRB |
| ZNF680 | | |

**Table S5 Pathological and clinical data of lung cancer cases in tissue microarray**

| Variables | Cases(n) (total n=92) | circDCUN1D4 | | P value |
|---|---|---|---|---|
| | | Low(n) | High(n) | |
| Age(yes) | | | | |
| ≤60 | 38 | 14 | 24 | 0.731 |
| >60 | 54 | 18 | 36 | |
| Gender | | | | |
| Male | 51 | 22 | 29 | 0.062 |
| Female | 41 | 10 | 31 | |
| Tumor size | | | | |
| ≤5cm | 69 | 23 | 46 | 0.618 |
| >5cm | 23 | 9 | 14 | |
| Lymph node invasion | | | | |
| Present | 41 | 15 | 26 | 0.008 |
| Absent | 51 | 17 | 34 | |
| TNM stage | | | | |
| I | 7 | 3 | 4 | 0.045 |
| II-Ⅲ | 85 | 29 | 56 | |
| EGFR mutation | | | | |
| Present | 22 | 9 | 13 | 0.495 |
| Absent | 70 | 23 | 47 | |

**Table S6 Univariate analysis and Multivariable analysis**

| Parameters | Univariate analysis | | | Multivariable analysis | | |
|---|---|---|---|---|---|---|
| | HR | 95%CI | P value | HR | 95%CI | P value |
| Age(>60) | 2.701 | 1.466-4.975 | 0.001 | 2.383 | 1.266-4.483 | 0.007 |
| Gender(Male) | 1.501 | 0.60-1.865 | 0.869 | — | — | — |
| Lymph node metastasis(Positive) | 3.297 | 1.857-5.855 | <0.0001 | 2.002 | 1.034-3.875 | 0.039 |
| Pathological grading(G2-G4) | 4.709 | 3.144-6.366 | <0.0001 | 3.805 | 2.540-5.091 | 0.002 |
| Tumor diameter(>5cm) | 4.150 | 2.185-7.884 | 0.031 | 1.676 | 0.756-3.716 | 0.204 |
| EGFR mutation(positive) | 4.563 | 2.427-8.577 | 0.215 | 1.405 | 0.636-3.107 | 0.401 |
| circDCUN1D4 level(low) | 2.250 | 1.285-3.940 | 0.005 | 1.593 | 1.205-2.922 | 0.033 |
