# Supplementary material for: circDCUN1D4 suppresses tumor metastasis and glycolysis in lung adenocarcinoma by stabilizing TXNIP expression
Source: Mol Ther Nucleic Acids. 2020 Nov 17;23:355–68. doi: 10.1016/j.omtn.2020.11.012 (PMC7779544; doi:10.1016/j.omtn.2020.11.012)
Supplement: Document S2. Article plus supplemental information [file mmc4.pdf]

Molecular Therapy
Nucleic Acids
Original Article

circDCUN1D4 suppresses tumor metastasis and glycolysis in lung adenocarcinoma by stabilizing TXNIP expression

Yingkuan Liang,[1,2,3,4,5] Hui Wang,[1,2,3,5] Bing Chen,[1,2,3,5] Qixing Mao,[1,2,3] Wenjie Xia,[1,2,3] Te Zhang,[1,2,3] Xuming Song,[1,2,3] Zeyu Zhang,[1,2,3] Lin Xu,[1,2,3] Gaochao Dong,[1,3] and Feng Jiang[1,2,3]

[1]The Affiliated Cancer Hospital of Nanjing Medical University, Nanjing, PR China; [2]Department of Thoracic Surgery, Jiangsu Cancer Hospital, Jiangsu Institute of Cancer Research, Nanjing 210029, PR China; [3]Jiangsu Key Laboratory of Molecular and Translational Cancer Research, Cancer Institute of Jiangsu Province, Nanjing, PR China; [4]Department of Thoracic Surgery, The First Affiliated Hospital of Suchow University, Suzhou, PR China

Aberrant expression of circular RNAs (circRNAs) is involved in cancer progression through interaction with RNA-binding proteins (RBPs). Herein, we screened circRNA expression of A549 cells in circBase and the crosslinking immunoprecipitation (CLIP) data of human antigen R (HuR), an extensively studied RBP, and identified a circRNA, circ-defective in cullin neddylation 1 domain containing 4 (circDCUN1D4), originating from the DCUN1D4 gene transcript. circDCUN1D4 is downregulated in tumor samples under the mediation of DExH-box helicase 9 (DHX9), which inhibits the formation of circRNA by binding inverted repeat Alus (IRAlus) in flanking sequences. circDCUN1D4 depletion promoted invasion *in vitro* and metastasis *in vivo*. Importantly, the interaction between circDCUN1D4 and HuR increased the transportation of HuR to the cytoplasm. circDCUN1D4 acts as a scaffold to facilitate the interaction between the HuR protein and thioredoxin-interacting protein (TXNIP) mRNA, which enhances the stability of the TXNIP mRNA. Additionally, circDCUN1D4 directly interacts with TXNIP mRNA through base complementation, indicating the formation of the circDCUN1D4/HuR/TXNIP RNA-protein ternary complex. Furthermore, circDCUN1D4 suppressed metastasis and glycolysis of lung cancer cells in a TXNIP-dependent manner. Clinically, the downregulated expression of circDCUN1D4 was more prevalent in lymph node metastatic tissues and served as an independent risk factor for the overall survival of lung adenocarcinoma (LUAD) patients. These findings demonstrated that a novel circRNA, circDCUN1D4, is involved in the metastasis and glycolysis of LUAD.

INTRODUCTION

Lung cancer is the most common cause of cancer-related death worldwide, and non-small cell lung cancer (NSCLC) is the most prevalent form, accounting for 85% of cases, of which lung adenocarcinoma (LUAD) is the most common subtype.[1] Advances in the treatment of NSCLC patients, such as surgery, radiation, chemotherapy, targeted therapies, and immune checkpoint inhibitor therapy, have been reported; however, the 5-year survival remains less than 15%.[2] The poor prognosis is due to the presence of locally advanced or metastatic tumors at the time of diagnosis in most patients.[3] Elucidation of the mechanism of tumor metastasis is urgently needed. Recently, noncoding RNAs (ncRNAs) have been defined as functional regulatory molecules instead of “junk” transcriptional products.[4] A previous report showed that microRNA (miR)-224/520c-dependent TUSC3 deficiency enhances the metastatic potential of NSCLC.[5] Circular (circ)TP63 promotes the progression of lung squamous cell carcinoma (LUSC) by competitively binding to miR-873-3p and upregulating FOXM1.[6] However, few studies have explored the mechanism of circRNAs in the metastasis of LUAD.

circRNAs, with a covalent single-stranded loop configuration, are produced by direct backsplicing or exon skipping of precursor mRNA (pre-mRNA).[7] circRNAs are stable and resistant to exonucleolytic RNA decay compared with their source mRNAs, and the structures have neither 5′–3′ polarity nor a polyadenylated tail; thus, these molecules have recently attracted increased research interest. Dysregulated expression of circRNAs has been identified in almost all types of cancers,[8] indicating that circRNAs have important noncoding functions. Many circRNAs have been proposed to act as microRNA (miRNA) sponges. ciRS-7/CDR1as harbors over 70 conventional miR-7 binding sites in neuronal tissues and is perhaps the best characterized circRNA.[9] However, several studies have shown that the majority of circRNAs harbor few binding sites for a single miRNA.[10] Recently, crosslinking immunoprecipitation (CLIP) datasets have shown the emerging roles of circRNAs in tumor progression via physical interactions with proteins; they act as protein sponges or

Received 25 June 2020; accepted 10 November 2020;
https://doi.org/10.1016/j.omtn.2020.11.012.

[5]These authors contributed equally

Correspondence: Gaochao Dong, The Affiliated Cancer Hospital of Nanjing Medical University, Baiziting 42, Nanjing 210009, PR China.
E-mail: ilsyvm@njmu.edu.cn
Correspondence: Feng Jiang, The Affiliated Cancer Hospital of Nanjing Medical University, Baiziting 42, Nanjing 210009, PR China.
E-mail: fengjiang_nj@njmu.edu.cn

This is an open access article under the CC BY license (http://creativecommons.org/licenses/by/4.0/).

scaffolds to mediate complex biological functions.[11] circNSUN2 interacts with the RNA-binding protein (RBP) IGF2BP2 to stabilize HMGA2, promoting colorectal liver metastasis.[12] circ-CUX1 binds to Ewing Sarcoma (EWS) RBP1 (EWSR1), resulting in transactivation of MYC-associated zinc finger protein (MAZ) and inhibiting glycolysis to suppress the progression of neuroblastoma.[13]

Human antigen R (HuR) is a member of the embryonic lethal abnormal visual (ELAV) protein family and is an extensively studied RBP that regulates protein expression patterns by associating with a wide range of RNAs.[14] Phosphorylated uridine diphosphate-glucose 6-dehydrogenase (UGDH) interacts with HuR and mediates the interaction of HuR with SNAI1 mRNA, which enhances the stability of SNAI1 mRNA and promotes lung cancer metastasis.[15] HuR is phosphorylated through p38 mitogen-activated protein kinase (MAPK), which results in cytoplasmic accumulation and enhances binding to $p21^{Cip1}$.[16] Many circRNAs were reported to bind HuR.[17] circ-AGO2 interacts with the HuR protein to facilitate its activation and enrichment on the 3′ untranslated region of target genes in gastric cancer.[18] However, whether circRNAs physically interact with HuR in LUAD is still unclear.

Glycolysis is one of the hallmarks of cancer and produces glucose-dependent ATP and glycolytic intermediates for macromolecular biosynthesis.[19] It has been reported that circ-CUX1 binds to EWSR1 and transactivates MAZ, which contributes to aerobic glycolysis and the progression of neuroblastoma.[13] Further, the circMAT2B/miR-338-3p/PKM2 axis promoted hepatocellular carcinoma progression by enhancing glycolysis.[20] It is not clear whether circRNAs participate in glycolysis during the progression of LUAD.

In this study, we identified a circ-DCUN1D4 RNA (circBase ID: hsa_circ_0007928), derived from the exon region of the DCUN1D4 gene, which is significantly downregulated in LUAD tissues. circDCU1ND4 interacted with HuR and facilitated HuR translocation to the cytoplasm, which suppressed glycolysis and metastasis in LUAD by stabilizing the TXNIP mRNA. Our results indicate that circDCUN1D4 may be a therapeutic target for the progression of LUAD.

RESULTS

Profiling of HuR-associated circRNAs and characterization of circDCUN1D4 in LUAD

To explore the circRNA interaction with the HuR protein in LUAD, we first analyzed the CLIP data of HuR[17] and identified 250 circRNAs (Figure 1A) associated with HuR. circRNA expression of A549 cells (Figure 1A) in the circRNA database circBase was also investigated to identify 14,970 circRNAs expressed in LUAD. Overlapping analysis with these circRNAs revealed that 152 circRNAs potentially interacted with HuR in LUAD (Data S1). According to the fold-enrichment rank, we identified the top 5 circRNAs expressed in the A549 cell line through quantitative reverse-transcriptase PCR (qRT-PCR) and found that hsa_circ_0007928 was highly expressed in A549 cells (Figure 1B). Furthermore, hsa_circ_0007928 was found to be downregulated in LUAD tumor tissues compared with matched adjacent normal tissues (Figure 1C). By mapping the sequences of hsa_circ_0007928 to the human reference genome (GRCh37/hg19), we revealed that hsa_circ_0007928 consists of exons 2, 3, 4, 5, and 6, derived from DCUN1D4; thus, we termed it as circDCUN1D4 (Figure 1D). The full-length sequence of circDCUN1D4 was also validated, and the sequence of circDCUN1D4 was similar to that in circBase (Figure S1A). The circDCUN1D4 (human) sequence and mmu_circDCUN1D4 (mouse) sequence were mapped, and the identities were 89%, which revealed that circDCUN1D4 is conserved (Figure S1B). The expression of circDCUN1D4 was measured in LUAD cell lines and the normal lung cell line HBE, which revealed that circDCUN1D4 was expressed at lower levels in the cancer cell lines than the normal cell line, and H1299, H1975, and A549 cells were chosen for use in the following experiments (Figure S1C). The cyclization of the 389-nucleotide (nt) circDCUN1D4 was validated by RT-PCR with Sanger sequencing (Figure 1D), RNase R treatment (Figures 1E and S1D), actinomycin D treatment (Figures 1F and S1E), and divergent primers (Figure 1G). Moreover, random hexamer or oligo $(dT)_{18}$ was used in RT experiments, and the relative expression of circDCUN1D4 was significantly decreased when oligo $(dT)_{18}$ primers were used (Figures 1H and S1F). This finding proved that circDCUN1D4 had no poly-A tail. Cytosolic/nuclear fractionation, followed by qRT-PCR analysis, revealed that circDCUN1D4 localized mainly in the cytosol (Figures 1I and S1G), which was confirmed by fluorescence *in situ* hybridization (FISH; Figure 1J). These results showed that circDCUN1D4 is downregulated and localizes in both the cytosol and nucleus.

The expression of circDCUN1D4 is regulated by DExH-box helicase 9 (DHX9) in LUAD

Recent research has shown that circRNA biogenesis is mainly modulated by both intronic complementary sequences (ICSs) and RBPs.[21,22] Therefore, we analyzed the ICSs of intron 1 and intron 6 by RepeatMasker, and we found five Alu sequences, which belong to the primate-specific short interspersed element (SINE) family of retrotransposons and are ~300 nt long. The intron 1 sequences contain two Alus—Alu and AluJo—whereas the intron 6 sequences contain three Alus, AluSc, and two AluSxs (Figure 2A). The orientations of these Alus are different. Based on the research showing that inverted repeat Alus (IR*Alus*) could potentially mediate cyclization of circRNA,[21,23] we hypothesized that AluJo in intron 1 and AluSc in intron 6 could play important roles in the biogenesis of circDCUN1D4. We constructed the mock, positive control, vector wild type (WT; AluJo + AluSc) and two deletion plasmids to test whether circDCUN1D4 was promoted by AluJo and AluSc (Figure 2B). After transfection with the six types of vectors, qRT-PCR showed that the deletion of AluJo or AluSc did not result in overexpression of circDCUN1D4 compared with that of the WT vector (Figure 2C), which revealed that AluJo and AluSc are needed for biogenesis of circDCUN1D4. Since RBPs recognize intronic sequences to promote circRNA biogenesis, we screened RBPs that have been reported to regulate the biogenesis of circRNAs, and we chose DHX9, and adenosine deaminase 1 acting on RNA (ADAR1) as candidates, which were reported to downregulate circRNAs. qRT-PCR assays showed that depletion of DHX9

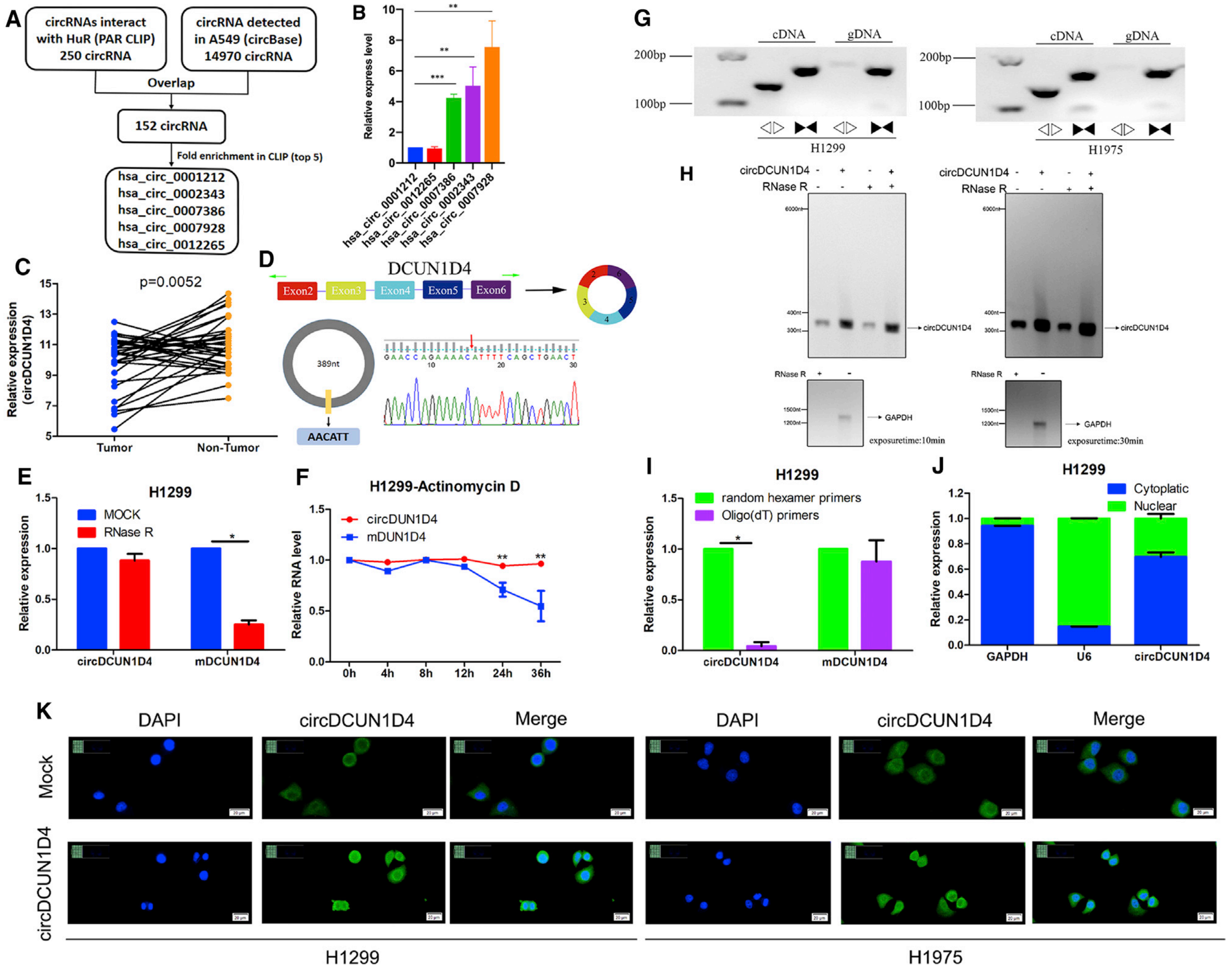

Figure 1. Expression profiles of circRNAs interacting with HuR in A549 cells and characterization of circDCUN1D4

(A) The flowchart delineating the identification of the top 5 circRNAs from CLIP data of HuR and A549 circRNA expression data from circBase. (B) The relative expression of the top 5 circRNAs in the A549 cell line (mean ± SD, n = 4). (C) qRT-PCR assay showing the relative expression of circDCUN1D4 (normalized to β-actin) in matched normal tissues and cancerous tissues of LUAD. (D) CircDCUN1D4 was backspliced by exons 2, 3, 4, 5, and 6 of the DCUN1D4 gene and validated by Sanger sequencing. (E) qRT-PCR for the abundance of circDCUN1D4 and DCUN1D4 in H1299 cells treated with RNase R compared with the mock cells (mean ± SD, n = 4). (F) qRT-PCR for the abundance of circDCUN1D4 and DCUN1D4 in H1299 cells treated with actinomycin D at the indicated time points (mean ± SD, n = 4). (G) RT-PCR or PCR assays for the detection of circDCUN1D4 using divergent and convergent primers from cDNA or genomic DNA (gDNA) of the LUAD cell lines H1299 and H1975. (H) Northern blot using a junction-specific probe indicating the endogenous existence. (I) Random hexamer or oligo (dT)18 primers were used in the reverse transcription experiment. The relative RNA levels were analyzed by qRT-PCR and normalized to the value using random hexamer primers (mean ± SD, n = 4). (J) qRT-PCR for the distribution of circDCUN1D4, GAPDH, and U6 in the cytoplasmic and nuclear fractions of cancer cells (mean ± SD, n = 4). (K) RNA-FISH assay showing the cytoplasmic and nuclear location of circDCUN1D4 in cultured H1299 and H1975 cells using a junction-specific antisense probe (green), with nuclear staining with DAPI (blue). Student's t test and analysis of variance compared the differences in (B, E, F, and I). *P<0.05, **P<0.001 vs. mock, mDCUN1D4, random hexamer primers. The Wilcoxon signed-rank test was used in (C).

significantly increased the expression of circDCUN1D4, whereas the knockdown of ADAR1 did not result in obvious changes (Figures 2D and S2A). DHX9 is an RNA helicase that unwinds IR*Alu* pairs.[25] Notably, the expression of circDCUN1D4 was negatively correlated with the expression of DHX9 in LUAD tissues, and DHX9 was obviously upregulated in LUAD tissues from The Cancer Genome Atlas (TCGA)[26] (Figures 2E and 2F). The RNA immunoprecipitation (RIP) assay was used to demonstrate the interaction between DHX9 and AluJo/AluSc (Figure 2G). According to the RNA-binding motif of DHX9 (Figure 2H), we screened two potential binding sites between DHX9 and AluJo/AluSc, respectively. The WT and mutation luciferase report plasmids of AluJo/AluSc were designed to validate the accurate binding site. The results released that DHX9 interacted with AluJo/AluSc in these two potential binding sites (Figure 2I). Thus, IRAlus could partly participate in the biogenesis of circDCUN1D4, which was mediated by DHX9.

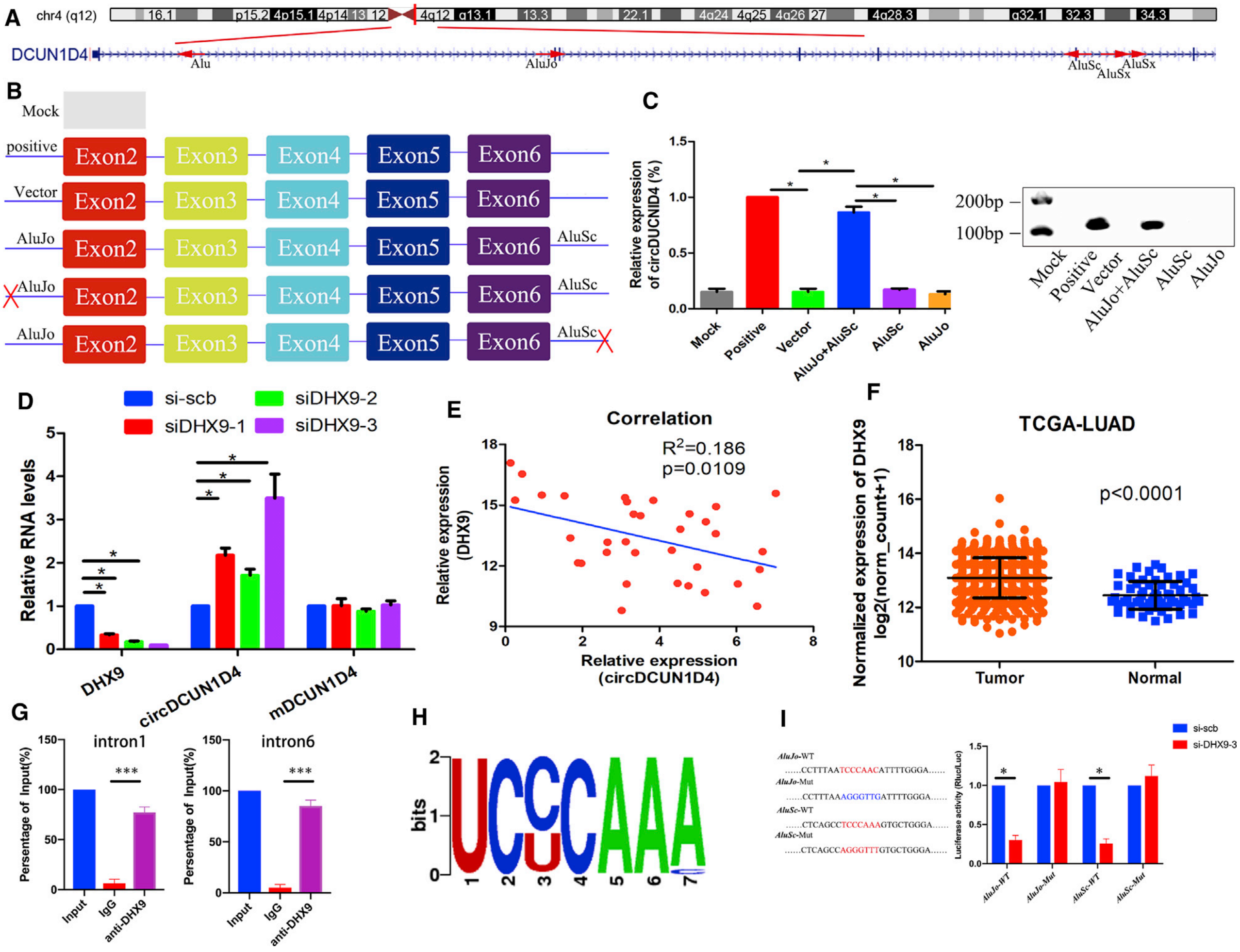

Figure 2. The expression of circDCUN1D4 in LUAD can be regulated by DHX9

(A) The location of DCUN1D4 in the genome and RepeatMasker analysis of the intron sequences of DCUN1D4. (B) A schematic drawing of six types of circDCUN1D4-overexpressing vectors. (C) qRT-PCR assay detected the expression of circDCUN1D4 after transfection with the six types of circDCUN1D4-overexpressing vectors (mean ± SD, n = 4). (D) qRT-PCR detection of circDCUN1D4 and mDCUN1D4 after DHX9 knockdown using RNAi in H1299 cells (mean ± SD, n = 4). (E) The correlation between the relative expression of DHX9 and circDCUN1D4 in 34 LUAD tissues. (F) The relative mRNA levels of DHX9 in LUAD in TCGA database. (G) The RIP assay results demonstrated that the DHX9 interacts with the *AluJo/AluSc* region (mean ± SD, n = 4). (H) The motif of DHX9. (I) The luciferase report assay results demonstrated that the DHX9 interacts with the *Alu* region of circDCUN1D4-intron1/intron6 (mean ± SD, n = 4).

circDCUN1D4 suppresses LUAD cell metastasis *in vitro* and *in vivo*

To evaluate the biological functions of circDCUN1D4 in LUAD, we first established circDCUN1D4-overexpressing and circDCUN1D4-knockdown LUAD cell lines by using a positive control vector (above-mentioned) or small hairpin RNAs (shRNAs) targeting the junction of circDCUN1D4 to transfect A549 cell lines and H1299 cell lines, respectively (Figures S2B and S2C). The expression of circDCUN1D4 changed significantly; however, the mDCUN1D4 and DCUN1D4 protein levels did not show obvious changes after overexpression or silencing of circDCUN1D4. Transwell assays, Matrigel assays, real-time cell analysis (RTCA), and wound-healing assays revealed that overexpressing circDCUN1D4 significantly suppressed the migration of LUAD cells (Figures 3A–3D), whereas knocking down circDCUN1D4 promoted the invasion of LUAD cells (Figures S2D–S2G). Immunofluorescence assays and western blotting also consistently showed that circDCUN1D4 overexpression increased the abundance of an epithelial marker (E-cadherin) and decreased the level of mesenchymal markers (N-cadherin and vimentin; Figures 3E, 3F, and S2H). Conversely, circDCUN1D4 silencing resulted in the opposite alterations.

To explore the effects of circDCUN1D4 *in vivo*, we injected athymic nude mice with circDCUN1D4-overexpressing A549 cells or negative control (NC) cells (mock) via the tail vein. Eight weeks later, circDCUN1D4-overexpressing cells resulted in fewer lung metastatic

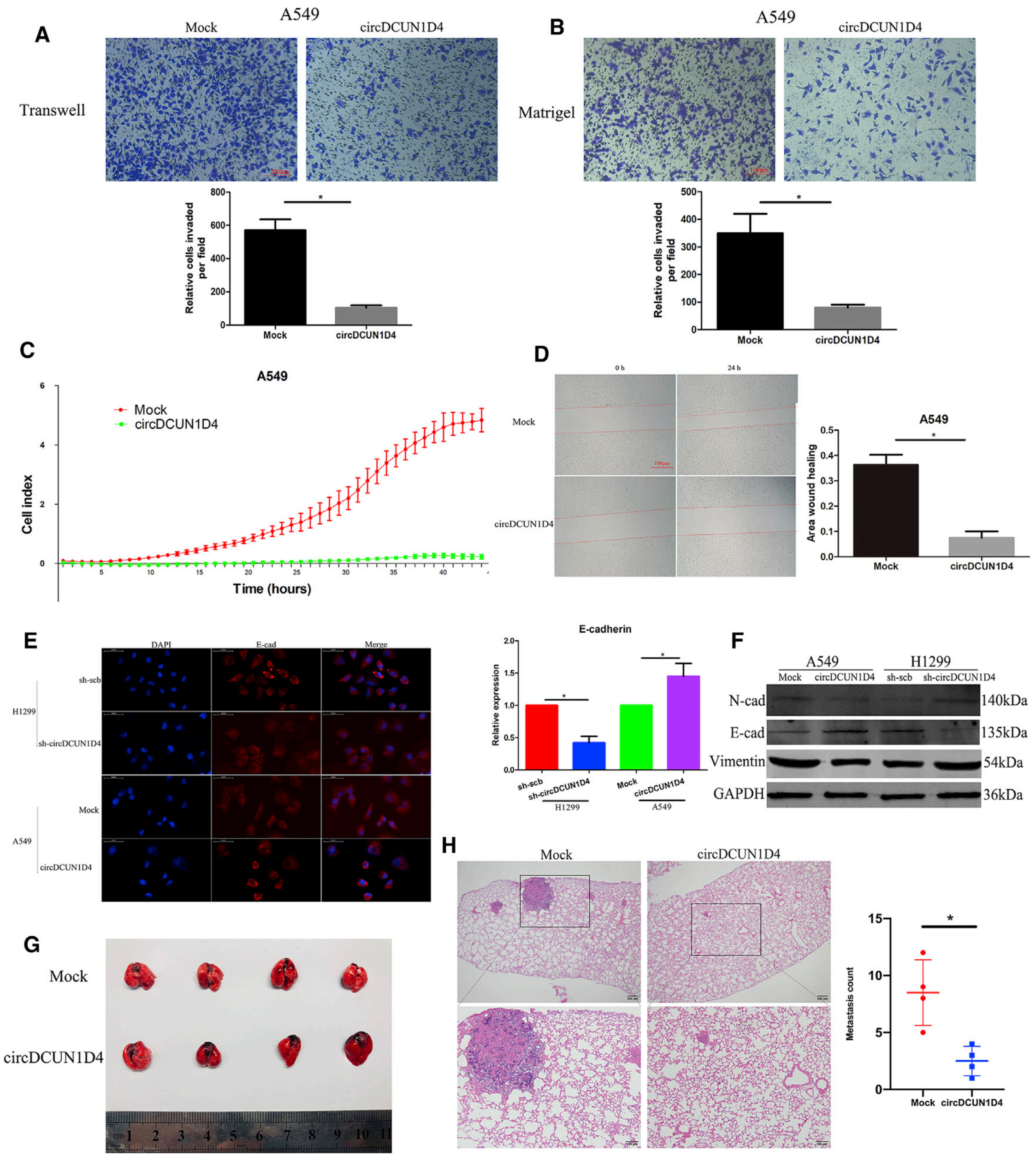

Figure 3. circDCUN1D4 suppresses the invasion and metastasis of cancer cells *in vitro* and *in vivo*

(A and B) Representative images (upper panel) and quantification (lower panel) of Transwell and Matrigel assays, respectively, showing the invasion of A549 cells stably transfected with empty vector (mock) and circDCUN1D4 (mean ± SD, n = 4). Scale bars, 100 μm. (C) Cell migration in real time was analyzed by the xCELLigence RTCA (mean ± SD, n = 4). (D) Representative images of the wound-healing assay showing the metastasis of A549 cells stably transfected with empty vector (mock) and circDCUN1D4 (mean ± SD, n = 4). Scale bar, 100 μm. (E) Representative images (left panel) and quantification (right panel) of the immunofluorescence staining assay showing the expression of E-cadherin (E-cad) in A549 and H1299 cells stably transfected with mock, circDCUN1D4, scramble shRNA (sh-scb), or sh-circDCUN1D4 vectors (mean ± SD, n = 4). (F) Western blot indicating the expression of N-cadherin (N-cad), E-cad, vimentin, and GAPDH in total lysates of A549 and H1299 cells stably transfected with mock, circDCUN1D4, sh-scb, or sh-circDCUN1D4 vectors. (G) Images of dissected mouse lungs after tail-vein injection of A549 cells stably transfected with mock and circDCUN1D4 vectors (n = 4 for each group). (H) H&E staining of pathological sections (left panel) and the quantification of metastasis count (right panel). Student's t test and analysis of variance compared the differences in (A–D) and (H). *$p < 0.05$ versus mock or sh-scb.

colonies than control cells (mock), and hematoxylin and eosin (H&E) staining of the dissected lungs confirmed that overexpression of circDCUN1D4 significantly suppressed lung metastasis (Figures 3G and 3H).

circDCUN1D4 promotes the cytoplasmic export of the HuR protein and activates HuR in cancer cells

To explore the interaction between circDCUN1D4 and the HuR protein, we first evaluated whether circDCUN1D4 interacted with HuR. Mfold[27] was utilized to identify optimal folding of the circDCUN1D4 secondary structure and then submitted to RNA Composer[28] to generate the 3D structure of circDCUN1D4. The 3D structure of the HuR protein was derived from Protein Data Bank (PDB: 4FXV). NPDock was then used to calculate the *in silico* molecular docking between circDCUN1D4 and HuR, which indicated that circDUCN1D4 could perfectly dock HuR (Figure 4A). RIP assays demonstrated the enrichment of circDCUN1D4 in complexes precipitated with antibody against HuR compared to those with control immunoglobulin G (IgG; Figure 4B). Further biotin-labeled circular or linear RNA pull-down and western blot assays demonstrated that circDCUN1D4 could physically interact with HuR (Figure 4C). Next, we studied which domain of HuR contributes to the interaction with circDCUN1D4. We constructed HuR mutants with truncation of individual protein domains, and RIP assays revealed that RNA recognition motif 1 (RRM1), not RRM2, RRM3, or the hinge domain, of HuR specifically bound to circDCUN1D3 (Figure 4D). Furthermore, we identified the critical motif of circDCUN1D4 for the interaction with HuR. We applied the Browser Extensible Data (BED) files belonging to HuR RBP-circRNA CLIP data downloaded from starBase and used an interactive graphical viewer (IGV) to visualize the peaks, which revealed that the motif contains AU-rich sequences located in exon 4 of circDCUN1D4 as a recognition element for HuR (Figures 4E and S3A).

We further investigated the interaction between circDCUN1D4 and HuR in regulating HuR expression. The mRNA and protein levels of HuR were not affected by overexpression or knockdown of circDCUN1D4, and the mRNA levels of circDCUN1D4 also were not affected by overexpression or knockdown of HuR (Figures S3B–S3D). However, cytosolic/nuclear fractionation, followed by western blot analysis and immunofluorescence assays, showed that overexpression of circDCUN1D4 facilitated HuR export from the nucleus to the cytoplasm, but knockdown of circDCUN1D4 also returned HuR back to the nucleus (Figures 4F, 4G, and S3E).

To evaluate the activity of HuR, we constructed dual-luciferase reporter minigenes that contain three canonical HuR binding sites located downstream of the Renilla luciferase. Overexpression or knockdown of circDCUN1D4 improved or suppressed the activity of HuR, respectively (Figure 4H). Taken together, these results demonstrated that circDCUN1D4 interacted with the HuR protein and facilitated the translocation of HuR to the cytoplasm, which promoted HuR activity.

circDCUN1D4 stabilizes TXNIP mRNA and suppresses glycolysis of LUAD through TXNIP

As HuR is essential for mRNA stability,[29] we explored the downstream targets stabilized by the circDCUN1D4/HuR complex using a mRNA microarray of A549 cells. There were 661 genes (Data S2) with 321 upregulated and 340 downregulated genes that were significantly differentially expressed (fold change > 2, adjusted p value [adj.p] < 0.05) upon circDCUN1D4 overexpression (Figure 5A). Among the 661 differentially expressed genes, 113 mRNAs bound by HuR were identified through the overlapping HuR CLIP sequence (CLIP-seq) dataset derived from starBase. Given that circDCUN1D4 suppresses the invasion of lung cancer, we screened 113 mRNAs that have been reported to be associated with cancer progression, and 19 genes were identified as candidate targets of circDCUN1D4 (Table S4). qRT-PCR assays revealed that the expression of TXNIP, cytochrome P450 family 2 subfamily J member 2 (CYP2J2), and glypican 3 (GPC3) changed significantly, both with overexpression of circDCUN1D4 and knockdown of circDCIN1D4 (Figures 5B and S3F). However, only the expression of TXNIP, not CYP2J2 or GPC3, was positively correlated with the expression of circDCUN1D4 in LUAD tumor tissues (Figures 5C and S3G), confirming that TXNIP is the target of circDCUN1D4. Kaplan-Meier Plotter was utilized to reveal that higher expression of TXNIP was associated with longer overall survival (OS) in the LUAD cohort (Figures 5D and S3H). We explored NCBI and found that the TXNIP gene has two transcripts: GenBank: NM_006472.6 and NM_001313972.2. We designed different primers to amplify different transcripts; TXNIP-1 was for the transcript GenBank: NM_006472.6, TXNIP-2 was for the transcript GenBank: NM_001313972.2, pre-TXNIP was for the pre-mRNA of TXNIP, and 3′ UTR was for the total transcripts of TXNIP (Figure 5E). qRT-PCR assays revealed that overexpression or knockdown of circDCUN1D4 increased or decreased the expression of both transcripts but not the pre-mRNA of TXNIP, which revealed that circDCUN1D4 regulated the expression of TXNIP at the post-transcriptional level (Figure 5F). Western blot analysis also confirmed the change in TXNIP associated with circDCUN1D4. We further found that overexpression of circDCUN1D4 maintained the mRNA stability of TXNIP (Figure 5G). Moreover, overexpression or knockdown of circDCUN1D4 increased or decreased the enrichment of HuR in the 3′ UTR of TXNIP (Figure 5H).

We further explored the interaction between circDCUN1D4 and TXNIP. BLAST analysis of the sequences showed that circDCUN1D4 directly targeted the 3′ UTR of TXNIP (Figure 5I). Further, the interaction between circDCUN1D4 and the 3′ UTR of TXNIP was confirmed by a pull-down assay (Figure 5I). The region of circDCUN1D4 that interacted with TXNIP was also located in exon 4. Based on the interaction motif, we constructed dual-luciferase reporter minigenes containing WT TXNIP-3′ UTR (TXNIP-WT) or mutant 3′ UTR (TXNIP-Mut), respectively. The results revealed that overexpression or knockdown of circDCUN1D4 dramatically increased or inhibited the luciferase activity of TXNIP-WT but not that of TXNIP-Mut (Figure 5I). Further, we investigated whether

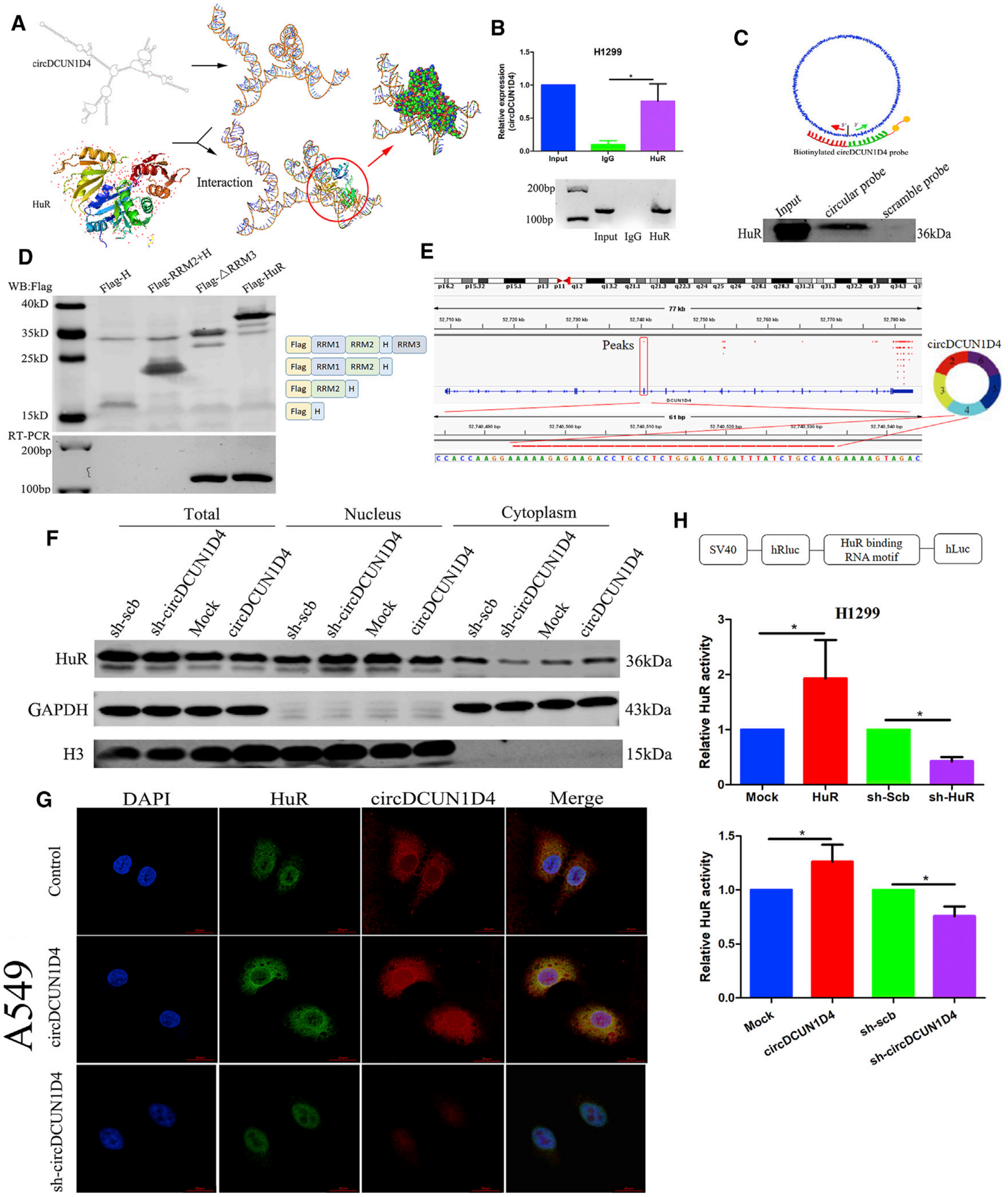

Figure 4. circDCUN1D4 interacts with and activates HuR protein in cancer cells

(A) Graphical representation of three-dimensional structures of circDCUN1D4 and HuR docking models with a zoom-in image of the binding interface generated by NPDock. (B) RIP assay showing that the RNA binding protein HuR interacts with circDCUN1D4. (C) RNA pull-down assay showing the HuR protein pulled down by circRNA probes or scramble probes from lysates of H1299 cells. (D) RIP assay depicting the recovered circDCUN1D4 levels from H1299 cells detected by RT-PCR (lower panel) after incubation with full-length or truncated forms of Flag-tagged recombinant HuR protein validated by western blot (upper panel). (E) IGV showing the peaks localized in exon 4 of circDCUN1D4, which interacts with the HuR protein. (F) Western blot indicating the expression of HuR in total lysates or subcellular fractions of H1299 cells stably transfected with mock, circDCUN1D4, sh-scb, or sh-circDCUN1D4 vectors. (G) Dual RNA-FISH and immunofluorescence staining assay showing the colocalization of circDCUN1D4 (red) and HuR (green) and the translocation of HuR (green) from the nucleus to the cytoplasm with DAPI (blue). Scale bar, 5 μm. (H) Dual-luciferase assay (middle and lower panels) indicating the activity of the HuR reporter (upper panel) in H1299 cells stably transfected with mock, HuR, circDCUN1D4, sh-scb, sh-HuR, or sh-circDCUN1D4 vectors (mean ± SD, n = 4). Student's t test compared the difference in (H). *$p < 0.05$ versus mock or sh-scb.

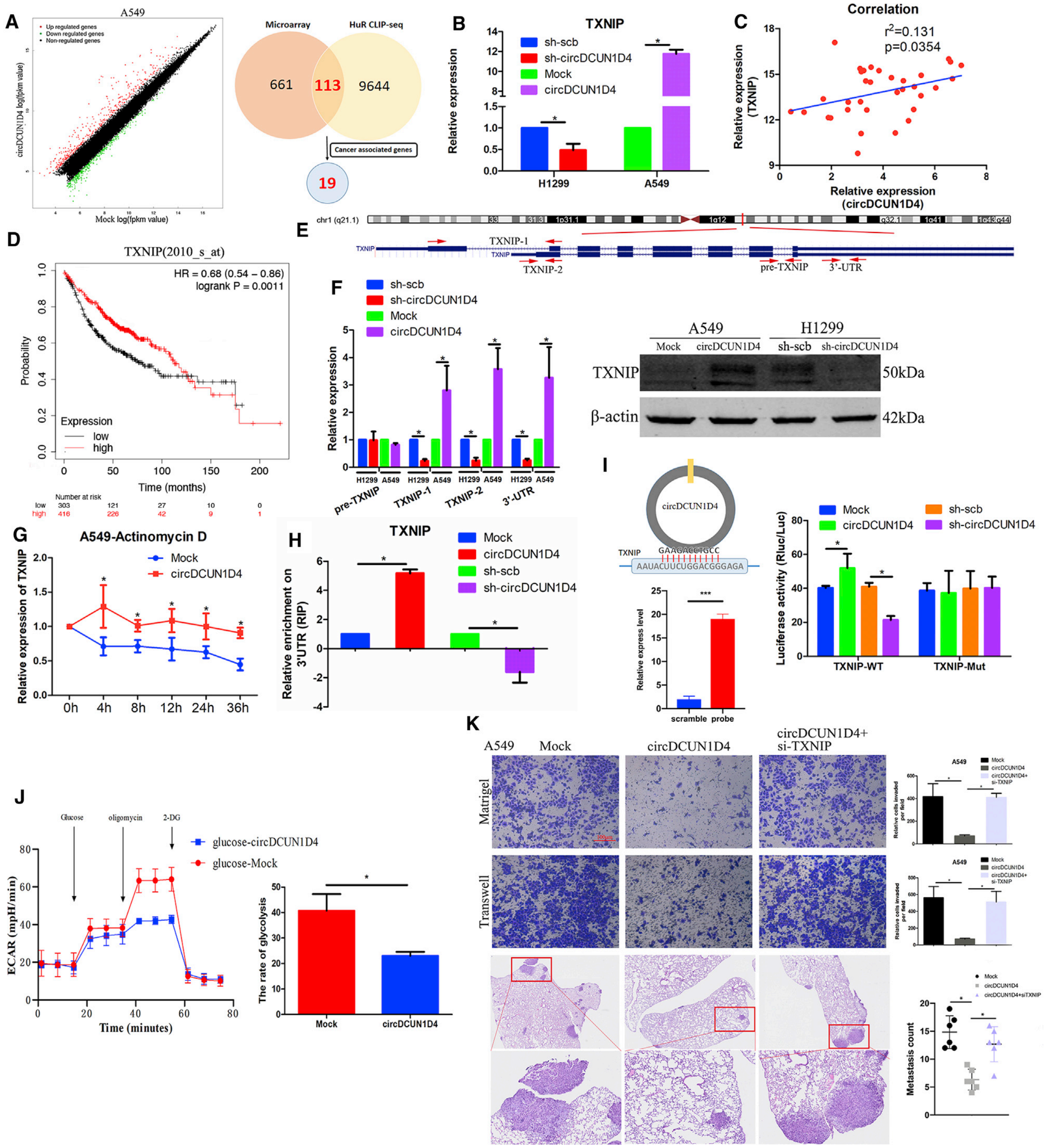

Figure 5. circDCUN1D4 allows HuR to stabilize TXNIP mRNA

(A) Microarray assay (left panel) showing the differentially expressed genes after stable transfection with mock and circDCUN1D4 vectors and a Venn diagram (right panel) showing the overlapping analysis of microarray results and HuR targets in the CLIPdb database. (B) qRT-PCR detection of TXNIP mRNA in A549 and H1299 cells stably transfected with mock, circDCUN1D4, sh-scb, or sh-circDCUN1D4 vectors (mean ± SD, n = 4). (C) The correlation between the relative expression of TXNIP and circDCUN1D4 in 34 LUAD tissues. (D) Kaplan-Meier curves indicating the overall survival (OS) of TXNIP in lung cancer cases derived from Kaplan-Meier Plotter. (E) The location of TXNIP in the genome (upper panel) and the designed primers determined by BLAST for TXNIP-1, TXNIP-2, pre-TXNIP, and 3′ UTR (lower panel). (F) qRT-PCR assay (left panel) detected the expression of pre-TXNIP, TXNIP-1, TXNIP-2, and 3′ UTR (mean ± SD, n = 4) and western blot assays (right panel), indicating the protein TXNIP

(legend continued on next page)

the suppression of circDCUN1D4 in metastasis is dependent on TXNIP. We designed TXNIP-overexpressed plasmid and short interfering RNAs (siRNAs; si-TXNIP-1, si-TXNIP-2, and si-TXNIP-3). We found that the plasmid can successfully overexpress TXNIP in A549 cells and that si-TXNIP-3 was the most efficient one to knock down TXNIP (Figure S4A). IGV also showed that the 3′ UTR of TXNIP contained many peaks that interacted with HuR, which were AU-rich elements (Figure S4B). Two potential binding sites between the 3′ UTR region of TXNIP and HuR were selected in starBase and were detected in more than three CLIP-seq datasets. Two WT luciferase reporter plasmids (WT-1 and WT-2) and relative mutation luciferase reporter plasmids (mutation-1 and mutation-2) were constructed to explore the binding site between the 3′ UTR region of TXNIP and HuR by the luciferase assay. The results released that the binding site was in Chr1: 145992937–145992958 (hg19) (Figure S4C). The expression level of TXNIP was significantly positive relative to the expression level of HuR in TCGA (Figure S4D). These results revealed that circDCUN1D4/HuR/TXNIP may form an RNA-protein ternary complex.

The degradation of TXNIP was reported to increase glycolysis to promote tumor cell migration.[30] We then explored whether the expression of circDCUN1D4 affected glycolytic activity in LUAD cells. The XF24 Extracellular Flux Analyzer (Seahorse) was utilized to analyze glycolysis and extracellular acidification rates (ECARs). The results revealed that overexpression of circDCUN1D4 inhibited glycolysis in A549 cells (Figure 5J). Further, we investigated whether the suppression of circDCUN1D4 in metastasis is dependent on TXNIP. Transwell, Matrigel, and nude mice tail-vein injection model assays demonstrated that the knockdown of TXNIP functionally rescued the decreased cell invasion and migration upon circDCUN1D4 overexpression (Figure 5K). In addition, knockdown of TXNIP and circDCUN1D4 reduced the cancer cell migration induced by circDCUN1D4 silencing (Figure S4E). These data indicated that circDCUN1D4 interacted with HuR to stabilize TXNIP and that circDCUN1D4 suppressed glycolysis and metastasis via TXNIP.

To explore whether circDCUN1D4 could act as a miRNA sponge, we performed an Ago2 reciprocal immunoprecipitation assay (Figure S4F). The results revealed that circDCUN1D4 barely adsorbed Ago2 protein compared to the NC (IgG) and CDR1as, which is believed to be exceptionally abundant in the brain and harbors more than 70 miR-7 binding sites.[9] Thus, circDCUN1D4 could not act as a miRNA sponge.

Downregulated circDCUN1D4 expression is associated with lymph node metastasis and predicts poor prognosis in LUAD patients

With the utilization of an LUAD tissue microarray (TMA) containing 92 paired LUAD tissues and matched normal tissues, we then assessed the correlation of circDCUN1D4 expression with clinicopathological characteristics by *in situ* hybridization (ISH). The results indicated that the level of circDCUN1D4 was significantly higher in the normal lung tissues than in the matched LUAD tissues (Figure 6A). Further analysis showed that the expression of circDCUN1D4 was significantly lower in the tissues from patients with lymph node-positive and TNM stage II–III LUAD than in those from patients with lymph node-negative and TNM stage I LUAD (Figure 6B; Table S5). Univariate analysis showed that age, lymph node metastasis, pathological grading, tumor size, epidermal growth factor receptor (EGFR) mutations, and circDCUN1D4 expression level were significantly correlated with OS (Table S6). Subsequently, multivariate analyses indicated that the expression level of circDCUN1D4 was an independent risk factor for OS (Figure 6C). Furthermore, Kaplan-Meier analysis showed that LUAD patients with low circDCUN1D4 expression had a shorter OS than those with high expression ($p = 0.0087$; Figure 6D). However, Kaplan-Meier Plotter revealed that the expression of mDCUN1D4 was not associated with patient outcomes ($p = 0.097$; Figure S4G). Taken together, these data indicated that the circDCUN1D4 expression level was negatively associated with metastasis and could be used as an independent prognostic factor for LUAD patients.

DISCUSSION

With the development of high-throughput sequencing technology and circRNA-specific computational tools,[31] circRNAs can be systematically detected.[32] Compared to their linear counterparts, circRNAs are stable due to their the covalently closed structure, and these molecules can be found in exosomes, urine, and plasma.[33,34] Further, the expression and diversity of circRNAs are tissue specific.[35] Recent studies have revealed that the biological functions of circRNAs are variable and are involved in microRNA inhibition, protein sponging, and the translation of some circRNAs.[36] In this study, we identified a novel circRNA, termed circDCUN1D4, which was downregulated in LUAD tissues compared with adjacent normal tissues. circDCUN1D4 may act as a scaffold to interact with the RBP HuR and TXNIP mRNA. The circDCUN1D4/HuR complex facilitated the transportation of HuR from the nucleus to the cytoplasm, which enhanced the stability of TXNIP mRNA.

expression of A549 and H1299 cells stably transfected with mock, circDCUN1D4, sh-scb, or sh-circDCUN1D4 vectors. (G) qRT-PCR assays detected the expression of TXNIP in A549 cells stably transfected with mock or circDCUN1D4 vectors and treated with actinomycin D at the indicated time points. (H) RIP assay depicting the recovered TXNIP levels from H1299 cells detected by qRT-PCR after stable transfection with mock, circDCUN1D4, sh-scb, or sh-DCUN1D4 vectors (mean ± SD, n = 4). (I, left upper panel) BLAST analysis showing that circDCUN1D4 directly targets the 3′ UTR of TXNIP with high AU content; (left lower panel) pull-down assay and (right panel) luciferase reporter assay showing the interaction between circDCUN1D4 and the 3′ UTR of TXNIP mRNA. (J) ECAR assay showing restoration of metabolic activity in A549 cells stably transfected with mock or circDCUN1D4. (K) Representative images (left panel) and quantification (right panel) of Transwell, Matrigel, and *in vivo* assays showing the invasion of A549 cells stably transfected with mock, circDCUN1D4, or circDCUN1D4 plus shTXNIP (mean ± SD, n = 4). Scale bar, 100 μm. Student's t test and analysis of variance compared the differences in (B), (F), (H), and (K). $^{*}p < 0.05$ versus mock, sh-scb, IgG, WT, and circDCUN1D4.

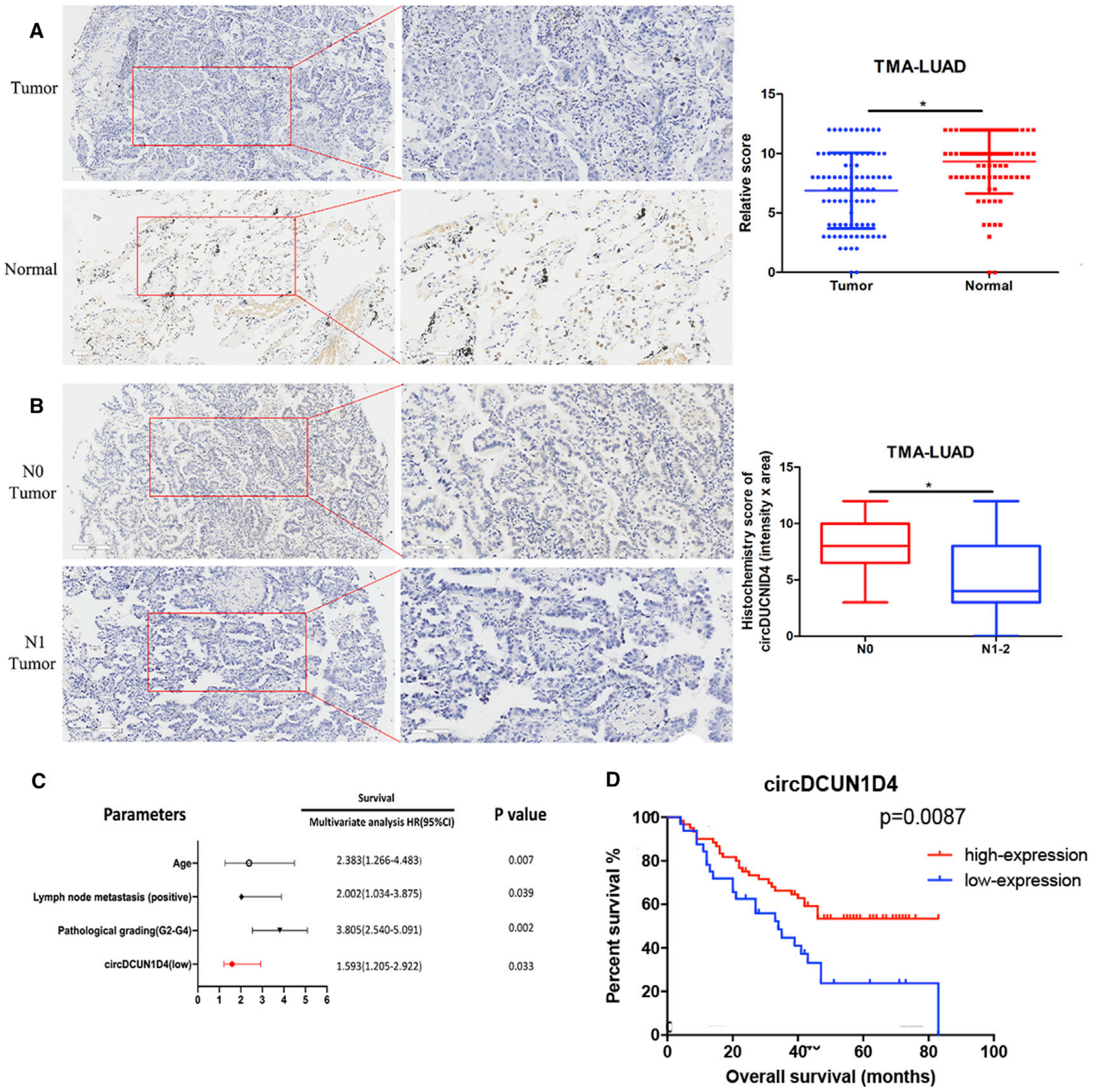

Figure 6. circDCUN1D4 is expressed at low levels and negatively associated with poor outcome in LUAD

(A) Representative images (left panel) and quantification of relative circDCUN1D4 expression between tumor and paired normal tissues detected by *in situ* hybridization (ISH) assay in LUAD tissue microarray (TMA). (B) Representative images (left panel) and quantification of relative circDCUN1D4 expression between N0 and N1–2 tumor tissues detected by ISH assays in LUAD TMA. (C) Multivariate analyses of hazard ratios for OS in LUAD TMA. (D) Kaplan-Meier analyses of correlations between circDCUN1D4 expression levels and OS among LUAD TMAs. Log-rank test for survival comparison in (D). Student's t test and analysis of variance compared the differences in (A) and (B). *p < 0.05 versus tumor and N0.

The biogenesis of circRNAs is a compelling research area; however, the mechanisms of circRNA biogenesis are still fairly elusive. circRNA formation can involve backsplicing and lariat formation; the former is the primary mechanism. Backsplicing often requires base pairing between inverted repeat elements (such as Alu elements), which are located in the intron sequences flanking the downstream splice donor site and the upstream splice acceptor site. Additionally, introns flanking the exons consisting of circRNAs tend to be long.[37] In this study, we analyzed flanking introns and identified two IRAlus located in the upstream and downstream introns: AluJo and AluSc. The deletion assay revealed that these two regions were critical for the formation of circDCUN1D4. The RBPs that bind to specific motifs or recognize double-strand RNA (dsRNA) can also facilitate circRNA production by binding to the molecule and stabilizing backsplicing. It has been reported that ADAR1 and DHX9 may suppress the biogenesis of circRNAs;[38] however, NF90 and NF110 can promote the production of circRNA by stabilizing intronic RNA pairs.[39] In this study, we showed that the lower expression of circDCUN1D4 was caused by upregulation of DHX9 in LUAD. DHX9, a nuclear RNA helicase, was shown to bind to IRAlus and unwound RNA pairs flanking

circularized exons, which inhibited circRNA expression.[25] However, the function of DHX9 in LUAD is unclear. Therefore, the biogenesis of circDCUN1D4 in LUAD also requires further exploration.

circRNAs can be classified into three categories based on their biogenesis: exonic circRNAs, intronic circRNAs, and exon-intron circRNAs. In the traditional view, the distribution of exonic circRNAs is mainly in the cytoplasm. Here, we found that circDCUN1D4, an exonic circRNA, was expressed in both the nucleus and cytoplasm. In the nucleus, circDCUN1D4 interacted with the RBP HuR. A recent study demonstrated that multiple RBPs not only were implicated in transcription control but also directly acted on chromatin acting as transcription factors (TFs).[40] It has been reported that HuR binds the CD133 promoter region and suppresses epithelial-mesenchymal transition in breast cancer.[41] The function of the circDCUN1D4/HuR complex in interacting with the DNA region to regulate transcription should be explored. In the cytoplasm, circDCUN1D4 can enhance the stability of TXNIP mRNA through dependence on the interaction between circDCUN1D4 and HuR. It is hypothesized that the ability of HuR to promote mRNA stabilization requires its translocation to the cytoplasm. In this study, we demonstrated that circDCU1N4 facilitated HuR cytoplasmic aggregation, which partly explained the shuttling of HuR between the nucleus and the cytoplasm. The function of HuR cytoplasmic accumulation of HuR is controversial. In resected pancreatic cancer, patients with HuR cytoplasmic accumulation may benefit from 5-fluorouracil (5-FU)-based adjuvant therapy and have good disease-free survival.[42] The tumor suppressor p161NK4a controls p21WAF1 induction by enabling the relocalization of HuR from the nucleus to the cytoplasm.[43] However, in glioblastoma cells, accumulation of HuR in the cytosol stabilizes the inflammatory cytokine interleukin-6, which contributes to promoting tumor progression and invasion.[44] In our study, we demonstrated that circDCUN1D4 promoted HuR translocation to the cytoplasm, which inhibited the metastasis and glycolysis in LUAD.

We also demonstrated that circDCUN1D4 could directly interact with the TXNIP mRNA through complementary bases, which implied that circDCUN1D4/HuR/TXNIP may form an RNA-protein ternary complex; this hypothesis requires further investigation. TXNIP, an important regulator of glucose and lipid metabolism, has attracted considerable attention based on its diverse functions on energy metabolism.[45,46] There are many factors that regulate the transcription of TXNIP. MYC stably represses TXNIP transcription;[47] in contrast, the MLX Interacting Protein/MAX-Like Protein X (MLXIP/MLX) complex upregulates the transcription of TXNIP by binding to the carbohydrate response element in the TXNIP promoter.[48] Additionally, the mRNA decay factor ZFP36 could directly target the TXNIP transcript to decrease its stability.[30] However, the regulation of TXNIP transcript stability has not been described in LUAD. Here, we provide evidence for acute regulation of TXNIP by circDCUN1D4 at the post-transcriptional level. Gain- and loss-of-function assays revealed that circDCUN1D4 inhibits metastasis, and ECAR assays showed that circDCUN1D4 decreases glycolysis, both of which depend on the TXNIP pathway.

In summary, our results suggest a protective role of circDCUN1D4 in LUAD by stabilizing TXNIP expression. Therefore, circDCUN1D4 may potentially be used in future treatment against LUAD, especially in patients with metastatic LUAD.

MATERIALS AND METHODS

Patient samples

Tumor tissues and paired normal lung tissues from LUAC patients who underwent surgery at the Department of Thoracic Surgery, Jiangsu Cancer Hospital (Nanjing, China), were collected and subjected to qRT-PCR analyses. All tumors and paired normal tissues were confirmed by pathologists. This study was approved by the Ethics Committee of Jiangsu Cancer Hospital in accordance with the ethical standards. All participants provided written, informed consent.

Cell culture

The human LUAC cell lines HBE, A549, H1299, H1975, PC-9, and SPCA-1 were purchased from ATCC and maintained in RPMI-1640 (KeyGen), except for SPCA-1, which was maintained in DMEM (KeyGen), and both media were supplemented with 10% fetal bovine serum (FBS; Life Technologies) at 37°C in a humidified atmosphere with 5% CO_2.

Bioinformatics analysis

Photoactivatable ribonucleoside (PAR) CLIP data from a previous article were downloaded,[17] and the circRNA expression profile of A549 was downloaded from circBase (http://www.circbase.org/). The Kaplan-Meier analysis was partly performed via Kaplan-Meier Plotter (http://www.kmplot.com/lung/). Interactions among circDCUN1D4, HuR, and TXNIP were analyzed via starBase version (v.)3.0 (http://starbase.sysu.edu.cn/index.php).

qPCR analysis

Total RNA was isolated using TRIzol reagent (Invitrogen). Quantification of circRNA and mRNA was carried out using PrimeScript RT Master Mix (TaKaRa). Before calculation using the ΔΔCt method, the levels of glyceraldehyde 3-phosphate dehydrogenase (GAPDH) or β-actin were used to normalize the relative expression levels of circRNAs and mRNAs, and the levels of small nuclear U6 were used to normalize the circRNA level in nuclear. The primers are provided in Table S1.

Western blotting analysis

Total protein was extracted from cells with radioimmunoprecipitation assay (RIPA) lysis buffer (Thermo Scientific) and PMSF (Beyotime), according to the manufacturer's instructions. The protein concentration was determined using a bicinchoninic acid (BCA) kit (KeyGEN). Comparable amounts of extracts were loaded on SDS-PAGE gels and subjected to electrophoresis. After separation on the gel, the proteins were transferred to a polyvinylidene fluoride (PVDF) membrane. Membranes were blocked in 2% BSA in Tris-buffered saline-Tween 20 (TBS-T) for 1 h and subsequently incubated overnight at 4°C, with antibodies against HuR (ab200342), TXNIP (ab188865), and H3 (ab1791), purchased from Abcam (Cambridge,

UK). Anti-Flag (#14793), anti-E-cadherin (#14472), anti-N-cadherin (#13116), anti-vimentin (#5741), anti-GAPDH (#5174), and anti-β-actin (#3700) were purchased from Cell Signaling Technology (Danvers, MA, USA) .

Overexpression or knockdown of genes

shRNAs targeting the junction region of the circDCUN1D4 sequence, circDCUN1D4-overexpressing plasmids, HuR-overexpressing plasmids, and TXNIP-overexpressing plasmids were synthesized by Hanbio (Shanghai, China). The primers are provided in Table S2. The siRNAs targeting TXNIP, DHX9, and ADAR1 were provided by RiboBio (Guangzhou, China). The target sequences are supplied in Table S3. The sequences of AluJo + AluSc, AluJo, AluSc, and vector were constructed by sequencing synthesis and subcloned into pcDNA3.1(+) (Public Protein/Plasmid Library, Nanjing, China). Transient transfection of the shRNA or the overexpressing plasmids was performed using the Lipofectamine 3000 kit (Invitrogen), according to the manufacturer's instructions, and transient transfection of siRNA was performed using the Lipofectamine iMax kit (Invitrogen), according to the manufacturer's instructions.

In vivo animal model and growth and metastasis assays

For *in vivo* metastasis assays, ten female BALB/c nude mice weighing 18–22 g were randomly assigned to two groups. A549 cells were prepared as a suspension of 0.4×10^6 cells in 200 μL of saline and inoculated into nude mice (5 mice per group) through the tail vein after transfection with sh-NC or sh-circDCUN1D4. After 8 weeks, the mice were killed, necropsies were carried out, and the lung metastatic nodules were counted. Staining with H&E confirmed that the nodules were metastatic tumors. The protocol used for these studies was approved by the Institutional Animal Care and Use Committee of the Affiliated Cancer Hospital of Nanjing Medical University. The animal study was carried out according to the State Food and Drug Administration of China's regulations on animal care. Animals were sorted only by treatment, and there was no exclusion or inclusion of an animal that was predetermined.

Biotin-labeled RNA pull-down

The biotin-labeled RNA probe of circDCUN1D4 was synthesized by GenePharma (Suzhou, China), and the sequence is provided in Table S2. In brief, lysates of 2×10^7 cancer cells were incubated with 3 μg of biotin-labeled linear or circRNA probe for 2 h and treated with 35 μL of Streptavidin C1 magnetic beads (Invitrogen) for 1 h. After three stringent washes, the retrieved protein was detected by western blots or qRT-PCR.

RNA immunoprecipitation assay

RNA immunoprecipitation experiments were performed using a Magna RIP RNA-Binding Protein Immunoprecipitation Kit (Millipore, USA), according to the manufacturer's instructions.

Statistics

All statistical analyses were performed with SPSS 25.0 software. Qualitative variables were analyzed by chi-square test or Fisher's exact test. For continuous variables that obey a normal distribution, Student's t test was used to compare the differences. Otherwise, variables were compared using a nonparametric test for which there was an abnormal distribution. Differences between groups were compared using analysis of variance (ANOVA) when applicable or a nonparametric test. Correlation analysis was performed using the Pearson correlation coefficient method. Receiver operating characteristic (ROC) curve analysis was performed to estimate the diagnostic sensitivity and specificity. Unless otherwise specified, the results are presented as the mean ± standard deviation (SD). All statistical tests were two-sided, and $p < 0.05$ was considered statistically significant.

SUPPLEMENTAL INFORMATION

Supplemental Information can be found online at https://doi.org/10.1016/j.omtn.2020.11.012.

ACKNOWLEDGMENTS

This study was approved by the Ethics Committee of the Nanjing Medical University Affiliated Cancer Hospital and was performed in accordance with the provisions of the Ethics Committee of Nanjing Medical University. All data generated or analyzed during this study are included in this published article and its additional information files. We thank the Department of Thoracic Surgery, Jiangsu Cancer Hospital, Nanjing, China, for providing the LUAD tissue samples and related anonymous clinical data. This study was supported by grants from the National Natural Science Foundation of China (grant numbers 81672294 and 81702892); Project of Invigorating Health Care through Science, Technology and Education, Jiangsu Provincial Medical Innovation Team (CXTDA2017002); Jiangsu Provincial Key Research and Development Program (BE2017761); Foundation of Jiangsu Cancer Hospital (ZK201601); and Young Talents Program of Jiangsu Cancer Hospital (23).

AUTHOR CONTRIBUTIONS

F.J. and G.D. conceived and designed the experiments. Y.L. and H.W. designed and carried out most of the experiments and manuscript writing. B.C., H.W., T.Z., X.S., and Z.Z. contributed to the experimental design, participated in the analysis of data, and helped in cell culture and sample collection. All authors reviewed and approved this manuscript.

DECLARATION OF INTERESTS

The authors declare no competing interests.

REFERENCES

1. Herbst, R.S., Morgensztern, D., and Boshoff, C. (2018). The biology and management of non-small cell lung cancer. Nature *553*, 446–454.

2. Jordan, E.J., Kim, H.R., Arcila, M.E., Barron, D., Chakravarty, D., Gao, J., Chang, M.T., Ni, A., Kundra, R., Jonsson, P., et al. (2017). Prospective Comprehensive Molecular Characterization of Lung Adenocarcinomas for Efficient Patient Matching to Approved and Emerging Therapies. Cancer Discov. *7*, 596–609.

3. Arbour, K.C., and Riely, G.J. (2019). Systemic Therapy for Locally Advanced and Metastatic Non-Small Cell Lung Cancer: A Review. JAMA *322*, 764–774.

4. Anastasiadou, E., Jacob, L.S., and Slack, F.J. (2018). Non-coding RNA networks in cancer. Nat. Rev. Cancer *18*, 5–18.

5. Jeon, Y.J., Kim, T., Park, D., Nuovo, G.J., Rhee, S., Joshi, P., Lee, B.K., Jeong, J., Suh, S.S., Grotzke, J.E., et al. (2018). miRNA-mediated TUSC3 deficiency enhances UPR and ERAD to promote metastatic potential of NSCLC. Nat. Commun. *9*, 5110.

6. Cheng, Z., Yu, C., Cui, S., Wang, H., Jin, H., Wang, C., Li, B., Qin, M., Yang, C., He, J., et al. (2019). circTP63 functions as a ceRNA to promote lung squamous cell carcinoma progression by upregulating FOXM1. Nat. Commun. *10*, 3200.

7. Chen, L.L. (2016). The biogenesis and emerging roles of circular RNAs. Nat. Rev. Mol. Cell Biol. *17*, 205–211.

8. Vo, J.N., Cieslik, M., Zhang, Y., Shukla, S., Xiao, L., Zhang, Y., Wu, Y.M., Dhanasekaran, S.M., Engelke, C.G., Cao, X., et al. (2019). The Landscape of Circular RNA in Cancer. Cell *176*, 869–881.e13.

9. Memczak, S., Jens, M., Elefsinioti, A., Torti, F., Krueger, J., Rybak, A., Maier, L., Mackowiak, S.D., Gregersen, L.H., Munschauer, M., et al. (2013). Circular RNAs are a large class of animal RNAs with regulatory potency. Nature *495*, 333–338.

10. Guo, J.U., Agarwal, V., Guo, H., and Bartel, D.P. (2014). Expanded identification and characterization of mammalian circular RNAs. Genome Biol. *15*, 409.

11. Kristensen, L.S., Andersen, M.S., Stagsted, L.V.W., Ebbesen, K.K., Hansen, T.B., and Kjems, J. (2019). The biogenesis, biology and characterization of circular RNAs. Nat. Rev. Genet. *20*, 675–691.

12. Chen, R.X., Chen, X., Xia, L.P., Zhang, J.X., Pan, Z.Z., Ma, X.D., Han, K., Chen, J.W., Judde, J.G., Deas, O., et al. (2019). N^6-methyladenosine modification of circNSUN2 facilitates cytoplasmic export and stabilizes HMGA2 to promote colorectal liver metastasis. Nat. Commun. *10*, 4695.

13. Li, H., Yang, F., Hu, A., Wang, X., Fang, E., Chen, Y., Li, D., Song, H., Wang, J., Guo, Y., et al. (2019). Therapeutic targeting of circ-CUX1/EWSR1/MAZ axis inhibits glycolysis and neuroblastoma progression. EMBO Mol. Med. *11*, e10835.

14. Lebedeva, S., Jens, M., Theil, K., Schwanhäusser, B., Selbach, M., Landthaler, M., and Rajewsky, N. (2011). Transcriptome-wide analysis of regulatory interactions of the RNA-binding protein HuR. Mol. Cell *43*, 340–352.

15. Wang, X., Liu, R., Zhu, W., Chu, H., Yu, H., Wei, P., Wu, X., Zhu, H., Gao, H., Liang, J., et al. (2019). UDP-glucose accelerates SNAI1 mRNA decay and impairs lung cancer metastasis. Nature *571*, 127–131.

16. Lafarga, V., Cuadrado, A., Lopez de Silanes, I., Bengoechea, R., Fernandez-Capetillo, O., and Nebreda, A.R. (2009). p38 Mitogen-activated protein kinase- and HuR-dependent stabilization of p21(Cip1) mRNA mediates the G(1)/S checkpoint. Mol. Cell. Biol. *29*, 4341–4351.

17. Abdelmohsen, K., Panda, A.C., Munk, R., Grammatikakis, I., Dudekula, D.B., De, S., Kim, J., Noh, J.H., Kim, K.M., Martindale, J.L., and Gorospe, M. (2017). Identification of HuR target circular RNAs uncovers suppression of PABPN1 translation by CircPABPN1. RNA Biol. *14*, 361–369.

18. Chen, Y., Yang, F., Fang, E., Xiao, W., Mei, H., Li, H., Li, D., Song, H., Wang, J., Hong, M., et al. (2019). Circular RNA circAGO2 drives cancer progression through facilitating HuR-repressed functions of AGO2-miRNA complexes. Cell Death Differ. *26*, 1346–1364.

19. Hanahan, D., and Weinberg, R.A. (2011). Hallmarks of cancer: the next generation. Cell *144*, 646–674.

20. Li, Q., Pan, X., Zhu, D., Deng, Z., Jiang, R., and Wang, X. (2019). Circular RNA MAT2B Promotes Glycolysis and Malignancy of Hepatocellular Carcinoma Through the miR-338-3p/PKM2 Axis Under Hypoxic Stress. Hepatology *70*, 1298–1316.

21. Pagliarini, V., Jolly, A., Bielli, P., Di Rosa, V., De la Grange, P., and Sette, C. (2020). Sam68 binds Alu-rich introns in SMN and promotes pre-mRNA circularization. Nucleic Acids Res. *48*, 633–645.

22. Li, X., Yang, L., and Chen, L.L. (2018). The Biogenesis, Functions, and Challenges of Circular RNAs. Mol. Cell *71*, 428–442.

23. Zhang, X.O., Wang, H.B., Zhang, Y., Lu, X., Chen, L.L., and Yang, L. (2014). Complementary sequence-mediated exon circularization. Cell *159*, 134–147.

25. Aktaş, T., Avşar Ilık, İ., Maticzka, D., Bhardwaj, V., Pessoa Rodrigues, C., Mittler, G., Manke, T., Backofen, R., and Akhtar, A. (2017). DHX9 suppresses RNA processing defects originating from the Alu invasion of the human genome. Nature *544*, 115–119.

26. Tomczak, K., Czerwińska, P., and Wiznerowicz, M. (2015). The Cancer Genome Atlas (TCGA): an immeasurable source of knowledge. Contemp. Oncol. (Pozn.) *19* (1A), A68–A77.

27. Zuker, M. (2003). Mfold web server for nucleic acid folding and hybridization prediction. Nucleic Acids Res. *31*, 3406–3415.

28. Popenda, M., Szachniuk, M., Antczak, M., Purzycka, K.J., Lukasiak, P., Bartol, N., Blazewicz, J., and Adamiak, R.W. (2012). Automated 3D structure composition for large RNAs. Nucleic Acids Res. *40*, e112.

29. Grammatikakis, I., Abdelmohsen, K., and Gorospe, M. (2017). Posttranslational control of HuR function. Wiley Interdiscip. Rev. RNA *8*, https://doi.org/10.1002/wrna.1372.

30. Sullivan, W.J., Mullen, P.J., Schmid, E.W., Flores, A., Momcilovic, M., Sharpley, M.S., Jelinek, D., Whiteley, A.E., Maxwell, M.B., Wilde, B.R., et al. (2018). Extracellular Matrix Remodeling Regulates Glucose Metabolism through TXNIP Destabilization. Cell *175*, 117–132.e21.

31. Salzman, J., Chen, R.E., Olsen, M.N., Wang, P.L., and Brown, P.O. (2013). Cell-type specific features of circular RNA expression. PLoS Genet. *9*, e1003777.

32. Szabo, L., and Salzman, J. (2016). Detecting circular RNAs: bioinformatic and experimental challenges. Nat. Rev. Genet. *17*, 679–692.

33. Li, Y., Zheng, Q., Bao, C., Li, S., Guo, W., Zhao, J., Chen, D., Gu, J., He, X., and Huang, S. (2015). Circular RNA is enriched and stable in exosomes: a promising biomarker for cancer diagnosis. Cell Res. *25*, 981–984.

34. Wu, Z., Sun, H., Liu, W., Zhu, H., Fu, J., Yang, C., Fan, L., Wang, L., Liu, Y., Xu, W., et al. (2020). Circ-RPL15: a plasma circular RNA as novel oncogenic driver to promote progression of chronic lymphocytic leukemia. Leukemia *34*, 919–923.

35. Conn, S.J., Pillman, K.A., Toubia, J., Conn, V.M., Salmanidis, M., Phillips, C.A., Roslan, S., Schreiber, A.W., Gregory, P.A., and Goodall, G.J. (2015). The RNA binding protein quaking regulates formation of circRNAs. Cell *160*, 1125–1134.

36. Xia, X., Li, X., Li, F., Wu, X., Zhang, M., Zhou, H., Huang, N., Yang, X., Xiao, F., Liu, D., et al. (2019). A novel tumor suppressor protein encoded by circular AKT3 RNA inhibits glioblastoma tumorigenicity by competing with active phosphoinositide-dependent Kinase-1. Mol. Cancer *18*, 131.

37. Jeck, W.R., Sorrentino, J.A., Wang, K., Slevin, M.K., Burd, C.E., Liu, J., Marzluff, W.F., and Sharpless, N.E. (2013). Circular RNAs are abundant, conserved, and associated with ALU repeats. RNA *19*, 141–157.

38. Ivanov, A., Memczak, S., Wyler, E., Torti, F., Porath, H.T., Orejuela, M.R., Piechotta, M., Levanon, E.Y., Landthaler, M., Dieterich, C., and Rajewsky, N. (2015). Analysis of intron sequences reveals hallmarks of circular RNA biogenesis in animals. Cell Rep. *10*, 170–177.

39. Li, X., Liu, C.X., Xue, W., Zhang, Y., Jiang, S., Yin, Q.F., Wei, J., Yao, R.W., Yang, L., and Chen, L.L. (2017). Coordinated circRNA Biogenesis and Function with NF90/NF110 in Viral Infection. Mol. Cell *67*, 214–227.e7.

40. Xiao, R., Chen, J.Y., Liang, Z., Luo, D., Chen, G., Lu, Z.J., Chen, Y., Zhou, B., Li, H., Du, X., et al. (2019). Pervasive Chromatin-RNA Binding Protein Interactions Enable RNA-Based Regulation of Transcription. Cell *178*, 107–121.e18.

41. Latorre, E., Carelli, S., Raimondi, I., D'Agostino, V., Castiglioni, I., Zucal, C., Moro, G., Luciani, A., Ghilardi, G., Monti, E., et al. (2016). The Ribonucleic Complex HuR-MALAT1 Represses CD133 Expression and Suppresses Epithelial-Mesenchymal Transition in Breast Cancer. Cancer Res. *76*, 2626–2636.

42. Tatarian, T., Jiang, W., Leiby, B.E., Grigoli, A., Jimbo, M., Dabbish, N., Neoptolemos, J.P., Greenhalf, W., Costello, E., Ghaneh, P., et al. (2018). Cytoplasmic HuR Status Predicts Disease-free Survival in Resected Pancreatic Cancer: A Post-hoc Analysis From the International Phase III ESPAC-3 Clinical Trial. Ann. Surg. *267*, 364–369.

43. Al-Mohanna, M.A., Al-Khalaf, H.H., Al-Yousef, N., and Aboussekhra, A. (2007). The p16INK4a tumor suppressor controls p21WAF1 induction in response to ultraviolet light. Nucleic Acids Res. *35*, 223–233.

44. Gurgis, F.M., Yeung, Y.T., Tang, M.X., Heng, B., Buckland, M., Ammit, A.J., Haapasalo, J., Haapasalo, H., Guillemin, G.J., Grewal, T., and Munoz, L. (2015). The p38-MK2-HuR pathway potentiates EGFRvIII-IL-1β-driven IL-6 secretion in glioblastoma cells. Oncogene *34*, 2934–2942.

45. Malone, C.F., Emerson, C., Ingraham, R., Barbosa, W., Guerra, S., Yoon, H., Liu, L.L., Michor, F., Haigis, M., Macleod, K.F., et al. (2017). mTOR and HDAC Inhibitors Converge on the TXNIP/Thioredoxin Pathway to Cause Catastrophic Oxidative Stress and Regression of RAS-Driven Tumors. Cancer Discov. *7*, 1450–1463.

46. Alhawiti, N.M., Al Mahri, S., Aziz, M.A., Malik, S.S., and Mohammad, S. (2017). TXNIP in Metabolic Regulation: Physiological Role and Therapeutic Outlook. Curr. Drug Targets *18*, 1095–1103.

47. Shen, L., O'Shea, J.M., Kaadige, M.R., Cunha, S., Wilde, B.R., Cohen, A.L., Welm, A.L., and Ayer, D.E. (2015). Metabolic reprogramming in triple-negative breast cancer through Myc suppression of TXNIP. Proc. Natl. Acad. Sci. USA *112*, 5425–5430.

48. Stoltzman, C.A., Peterson, C.W., Breen, K.T., Muoio, D.M., Billin, A.N., and Ayer, D.E. (2008). Glucose sensing by MondoA:Mlx complexes: a role for hexokinases and direct regulation of thioredoxin-interacting protein expression. Proc. Natl. Acad. Sci. USA *105*, 6912–6917.

OMTN, Volume *23*

Supplemental Information

circDCUN1D4 suppresses tumor metastasis and glycolysis in lung adenocarcinoma by stabilizing TXNIP expression

Yingkuan Liang, Hui Wang, Bing Chen, Qixing Mao, Wenjie Xia, Te Zhang, Xuming Song, Zeyu Zhang, Lin Xu, Gaochao Dong, and Feng Jiang

Supplementary Materials and Methods

Actinomycin D and RNase R treatment

Cells were planted into six-well plates. Up to 60% confluency after 24 h, cells were treated with 5μg/ml Actinomycin D at indicated time points.

Total RNA (2μg) was incubated with 3 U/μg of RNase R (Sigma) for 15 min at 37 °C. After treatment with Actinomycin D or RNase R, the RNA expression levels of circDCUN1D4 and other mRNAs were analyzed by qRT-PCR.

Transwell migration and invasion assay

4×10^4 cells, suspended in medium without FBS, were seeded into transwell chambers (Corning), with or without Matrigel (Sigma-Aldrich) coating. The lower chamber contained medium with 10% FBS as chemokine. Twenty-four hours later, the migratory or invasive cells on the lower surface of the chamber were photographed and counted in 10 random microscopic fields after crystal violet staining.

Wound-healing assay

The transfected cells were cultured in 6-well plates. After the cells reached 90% confluence, a standard 200μl pipette tip was subsequently utilized to scratch linear wounds. In addition, the cell monolayers were cultivated in FBS-free medium. After scratching, the images of the wound closure were captured at 0, and 24h.

Real-time cell analysis

The CIM-plate16 contains 16 wells, each a modified Boyden chamber, which can be used independently but simultaneously to measure cell migration in real-time through 8μm pores of a polyethylene terephthalate membrane on to gold electrodes on the

underside of the membrane using the xCELLigence system (ACEA Biosciences, USA). Experiments were set up according to the manufacturer's instructions with the membrane uncoated (migration) or coated with growth-factor-reduced-matrigel (invasion) (BD BioSciences, UK) (20 μl 1:40 diluted matrigel per well on the upper surface). Cell index (electrical impedance) was monitored every 30 min for the duration of the experiment. Traces show the average of quadruplicate wells.

RNA-Fluorescence in situ hybridization assay and Fluorescence immunocytochemical staining

RNA-Fluorescence in situ hybridization (FISH) assays were performed using a RNA-FISH kit (GenePharma, China) according to the manufacturer's instructions. Cy3-labeled antisense probe was synthesized by GenePharma company (Suzhou, China) against circDCUN1D4. The sequence is listed in Table S2. In briefly, A549 cells were fixed with 4% paraformaldehyde. After pre-hybridization with 1× PBS/0.5% Triton X-100, cells were blocked and hybridized in hybridization buffer with Cy3-labeled antisense probe at 37 °C overnight. Then cells were incubated with specific antibodies for HuR (ab200342, 1:200 dilution) at 4 °C overnight. Cells were treated with tyramide-conjugated goat anti-rabbit IgG (Invitrogen 1:10000 dilution) and DAPI (300 nmol/L) staining. The images were photographed under a Nikon A1Si Laser Scanning Confocal Microscope (Nikon Instruments Inc, Japan).

Luciferase reporter assay

Human HuR luciferase reporter was constructed according to the previous article[2]. The TXNIP binding sites of circDCUN1D4 were analyzed by StarBase

(http://starbase.sysu.edu.cn/index.php). The different fragment sequences were synthesized and then inserted into the psiCHECK-2 vector (Promega). All vectors were verified by sequencing and luciferase activity was assessed using the Dual Luciferase Assay Kit (Promega) according to the manufacturer's instructions. The sequences were provided in Table S2

Microarray assay

The Agilent human mRNA array was designed with 8 identical arrays per slide (8 × 60 K format), with each array containing probes interrogating approximately 27958 Entrez Gene RNAs and 7419 long intergenic noncoding RNA. The array also contains 1280 Agilent control probes. Total RNA containing small RNA was extracted from cells by using TRIzol reagent (Invitrogen) according to the manufacturer's protocol. The purity and concentration of RNA were determined from optical density 260/280 readings using a spectrophotometer (NanoDrop ND-1000). RNA integrity was determined by 1% formaldehyde denaturing gel electrophoresis.

Tissue microarray and in situ hybridization assay

Tissue microarray (TMA) was obtained from Outdo Biotech Co. Ltd. (Shanghai, China). Ninety-two pairs of LUAC tissues and their paired peripheral normal lung tissues were used to construct the TMA. Special biotin labelled probe against circDCUN1D4 was synthesized by GenePharma company (Suzhou, China). The sequence is provided in Table S2. After incubating with the biotin labelled probe, the TMA was then stained with DAB and hematoxylin, dehydrated and covered. The TMA staining scores were evaluated by two independent observers blinded to the clinicopathological data. The

staining scores were based on two indicators: the proportion of positively stained cells and the staining intensity. The proportion of positively stained cells was evaluated with five scoring levels: 0, <10%; 1, 10–25%; 2, 25–50%; 3, 50–75%; and 4, >75%. The staining intensity was scored with the following point system: 0 (no staining), 1 (yellow), 2 (yellow-brown) and 3 (dark brown). The products of the above two indicators were considered the total score.

Files uploaded separately in Excel Format:

Additional Dataset 1. Fold enrichment of 152 circRNA in HuR CLIP.

Additional Dataset 2. 661 genes altered by circDCUN1D4.

Supplementary Figures

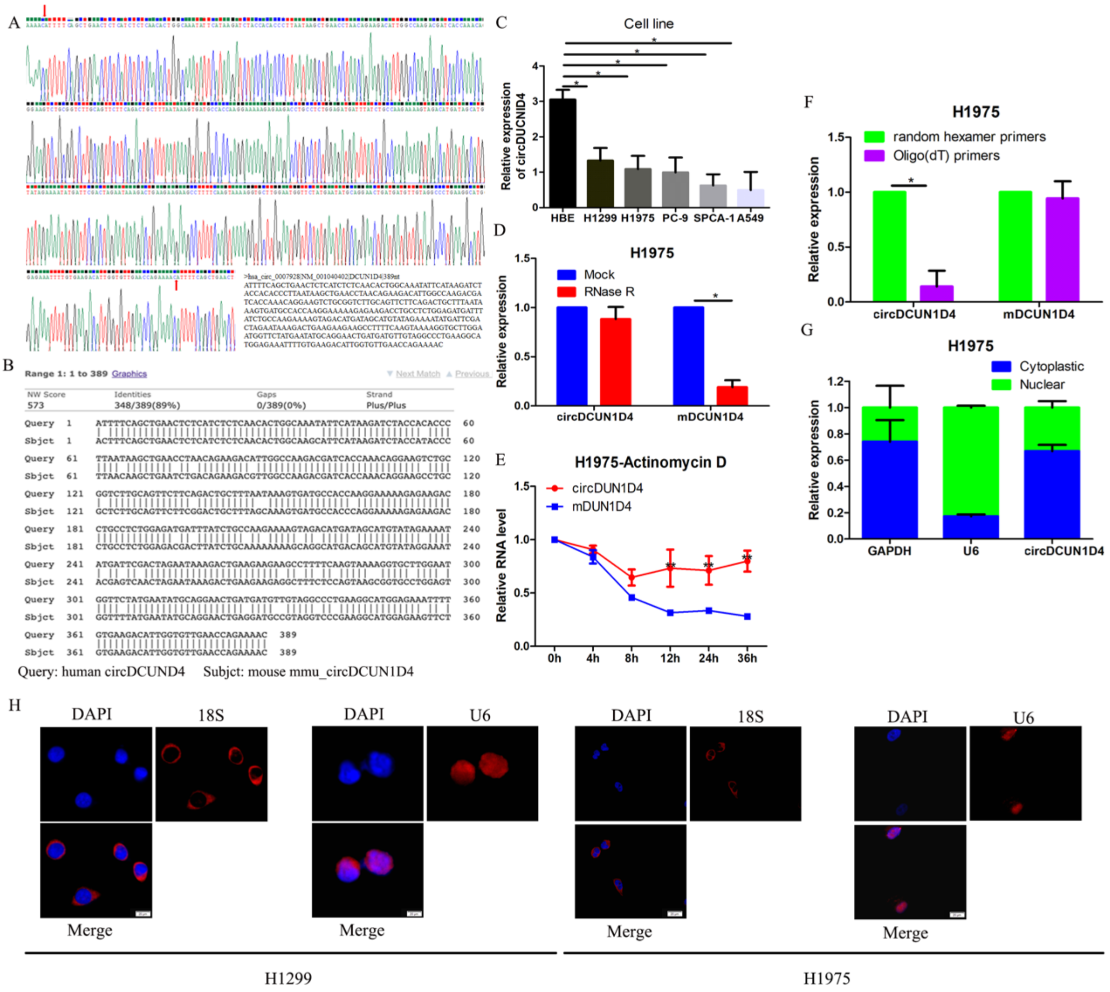

Fig. S1 Characterization of circDCUN1D4. A The full length of circDCUN1D4. **B** Conservative analysis of circDCUN1D4 between humans and mice. **C** qRT-PCR assay detected the expression of circDCUN1D4 in a variety of LUAD cell lines and a normal lung cell line HBE. (mean ± SD, n = 4) **D** qRT-PCR for the abundance of circDCUN1D4 and DCUN1D4 in H1975 cells treated with RNase R compared with the mock group. (mean ± SD, n = 4) **E** qRT-PCR for the abundance of circDCUN1D4 and DCUN1D4 in H975 cells treated with Actinomycin D at the indicated time point. (mean ± SD, n = 4) **F** Random hexamer or oligo (dT)18 primers were used in the reverse transcription experiment. The relative RNA levels were analyzed by qRT-PCR and normalized to the value using random hexamer primers. (mean ± SD, n = 4) **G** qRT-PCR for the distribution of circDCUN1D4, GAPDH, and U6 in the cytoplasmic and nuclear fractions of H1975 cells. **H** The 18S and U6 were applied as positive control for

cytoplasm and nucleus respectively in RNA-FISH assay. Student's t test and analysis of variance compared the difference in C, D, and E. *P < 0.05 vs. HBE, mock, mDCUN1D4.

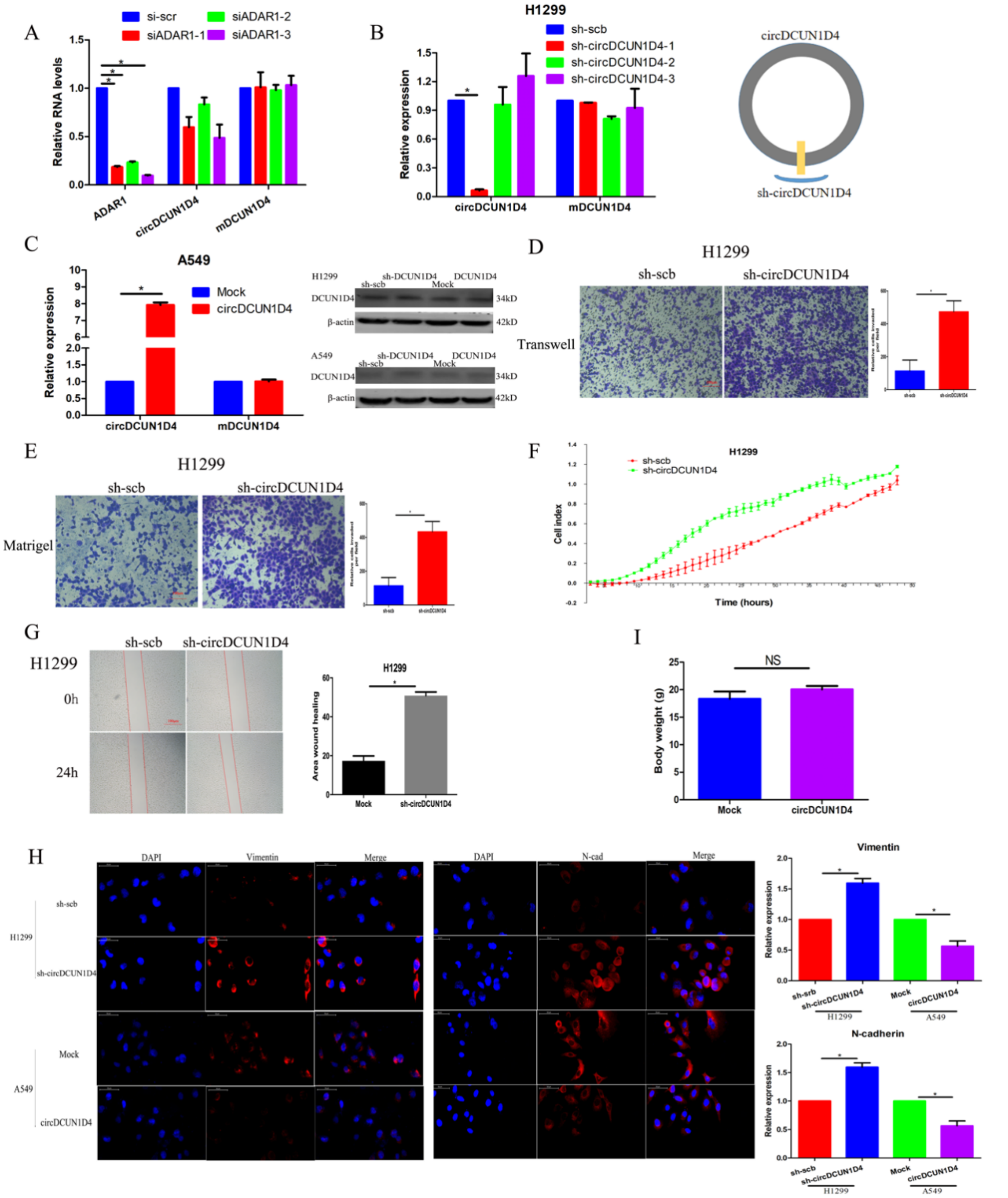

Fig. S2 Effects of circDCUN1D4 on the invasion of cancer cells. A qRT-PCR assay showing the transcript levels of EIF4A3, QKI, and ADAR1 in cancer cells stably transfected with si-scb, or siEIF4A3, or siQKI, or siADAR1, respectively. (mean ± SD, n = 4) **B** qRT-PCR and western blot assays showing the transcript and protein levels of DCUNID4 in cancer cells stably transfected with mock, circDCUN1D4, sh-Scb, or sh-DCUN1D4. (mean ± SD, n = 4)**C-F** Representative images and quantification of transwell (C), matrigel (D), C-plate (E) and wounding healing (F) assays showing the invasion of H1299 cells stably transfected with sh-scb or sh-DCU1ND4 (mean ± SD,

n=4). Scale bar: 100 μm. **G** Representative images (left panel) and quantification (right panel) of immunofluorescence staining assay showing the expression of Vimentin or N-cadherin of A549 and H1299 cells stably transfected with mock, circDCUN1D4, scramble shRNA (sh-scb), or sh-circDCUN1D4 (mean ± SD, n=4). **H** The body weight of mice after tail vein injection of A549 cells stably transfected with mock and circDCUN1D4 (n=4 for each group). Student's t test and analysis of variance compared the difference in A-G. *P < 0.05 vs. si-scb, mock, sh-scb.

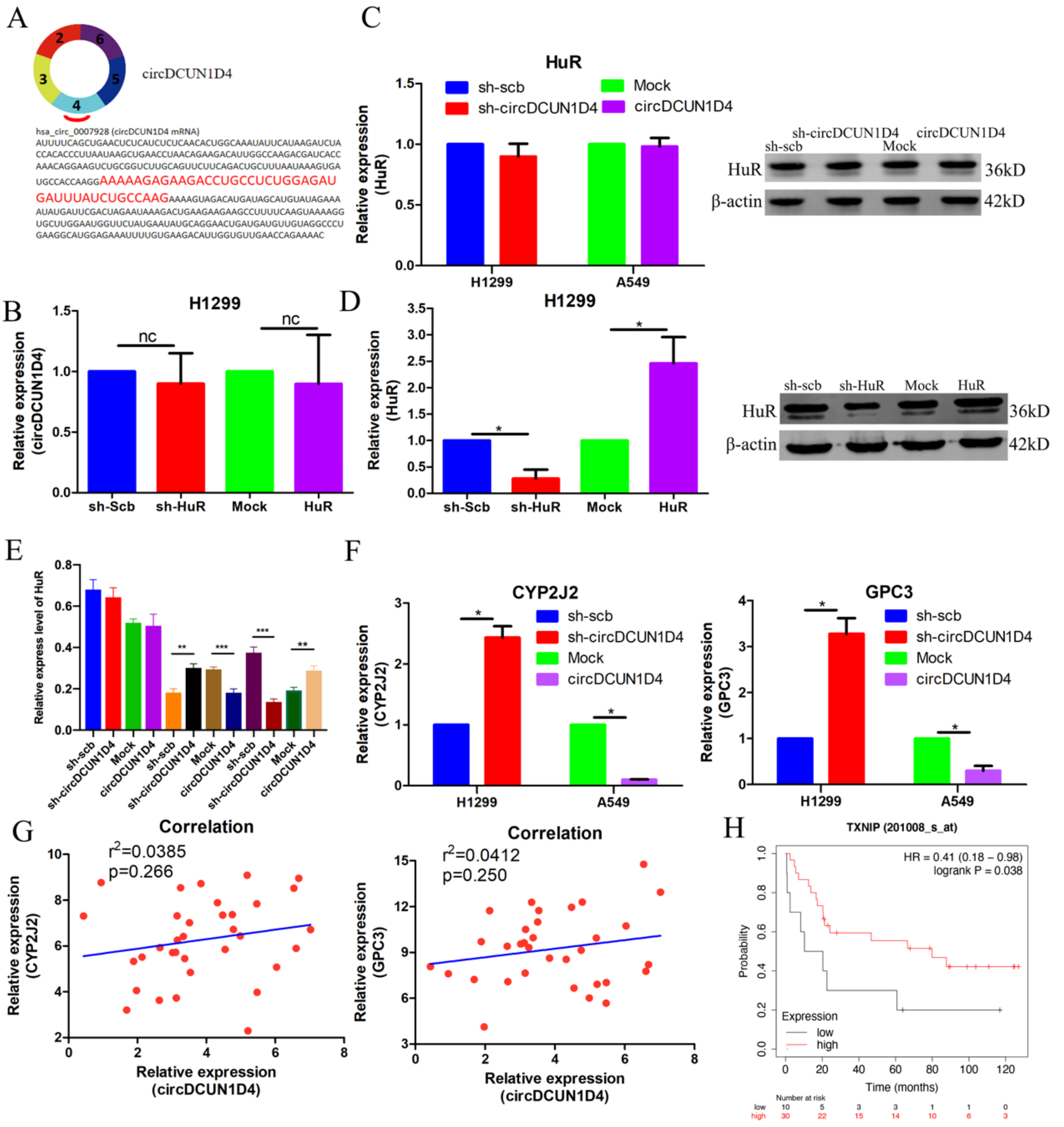

Fig. S3 Effects of circDCUN1D4 on the expression of HuR, CYP2J2 or GPC3 in cancer cells. A The sequences of circDCUN1D4 interacts with HuR protein. **B** qRT-PCR assay showing the expression of circDCUN1D4 in cancer cells stably transfected with mock, HuR, sh-scb, or sh-HuR. (mean ± SD, n=4) **C** qRT-PCR and western blot assays showing the transcript and protein levels of HuR in cancer cells stably transfected with mock, circDCUN1D4, sh-Scb, or sh-DCUN1D4. (mean ± SD, n=4) **D** qRT-PCR and western blot assays showing the transcript and protein levels of HuR in cancer cells stably transfected with mock, HuR, sh-scb, or sh-HuR. (mean ± SD, n=4) **E** The quantification of Western blot indicating the expression of HuR in total lysates

or subcellular fractions of H1299 cells stably transfected with mock, circDCUN1D4, sh-scb, or sh-circDCUN1D4 vectors. **F** qRT-PCR detection of CYP2J2 or GPC3 in cancer cells stably transfected with mock, circDCUN1D4, scramble shRNA (sh-scb), or sh-circDCUN1D4 (mean ± SD, n=4). **G** The correlation between the relative expression of CYP2J2 or GPC3 and circDCUN1D4 in 34 LUAD tissues. **H** Kaplan-Meier curves indicating the overall (OS) survival of TXNIP in lung cancer cases derived from Kaplam-Meier Plotter. Student's t test and analysis of variance compared the difference in A-F. ***P<0.001, **P<0.01, *P < 0.05 vs. mock, sh-scb.

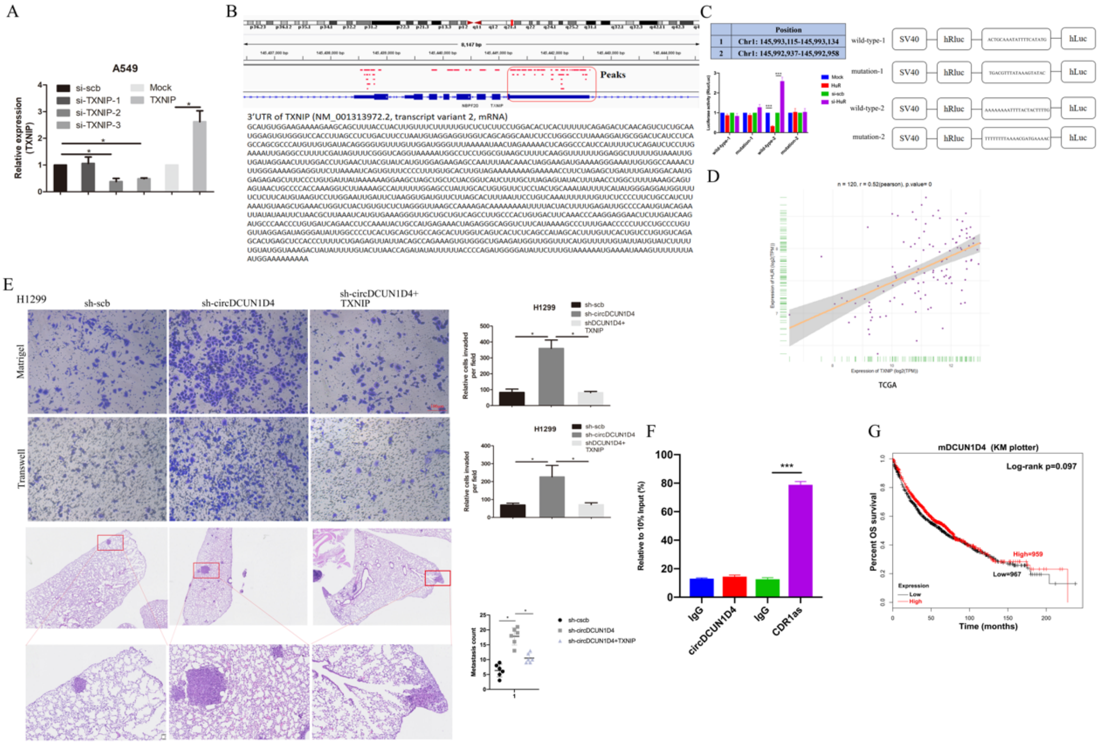

Fig. S4 circDCUN1D4 suppresses metastasis depend on TXNIP pathway. A qRT-PCR assay showing the expression of TXNIP in cancer cells stably transfected with Mock, TXNIP, si-scb, si-TXNIP-1, si-TXNIP-2, and si-TXNIP-3. (mean ± SD, n=4). **B** IGV showing the peaks localized in 3'UTP of TXNIP which interacts with HuR protein. **C** Dual-luciferase assay indicating the interacted region between 3'UTR region of TXNIP and HuR in H1299 cells stably transfected with mock, HuR, si-scb, or si-HuR (mean ± SD, n=4). **D** The expression level of TXNIP was significantly positive relative to the expression level of HuR. **E** Representative images (left panel) and quantification (right panel) of transwell, matrigel, and *in vivo* assays showing the invasion of H1299 cells stably transfected with sh-scb, sh-circDCUN1D4 or sh-circDCUN1D4 plus TXNIP (mean ± SD, n=4). Scale bar: 100 μm. **F** RIP assay showing that the RNA binding protein Ago2 could not interacts with circDCUN1D4. **G** Kaplan-Meier curves indicating the overall (OS) survival of DCUN1D4 in lung cancer cases derived from Kaplam-Meier Plotter. Student's t test and analysis of variance compared the difference in A, E and F. ***P<0.001, *P < 0.05 vs. mock, sh-scb, sh-circDCUN1D4, AND IgG. Log-rank test for survival comparison in G.

Supplementary Tables

Supplementary Table S1 Primer sets used for RT-PCR, qRT-PCR, and RIP

Primer set	Primers	Sequence	Application
hsa_circ_0001212	Forward	5’ -GTGAAGCGAGACAGTGGTGA-3’	qRT-PCR
	Reverse	5’ -ACCACAACCAGCTGACAAAA-3’	
hsa_circ_0002343	Forward	5’ -GTGGTGTGCAGGTGAACAAG-3’	qRT-PCR
	Reverse	5’ -ACAACACGCCAACTACCACA-3’	
hsa_circ_0007386	Forward	5’ -CCCGGACAGCTATGAAACTC-3’	qRT-PCR
	Reverse	5’ -AGGAGCATAACCCTCGATCA-3’	
hsa_circ_0007928 （circDCUN1D4）	Forward	5'- GTTGAACCAGAAAACATTTTC-3	qRT-PCR, RIP RT-PCR
	Reverse	5'- AGACTTCCTGTTTGGTGATC -3'	
hsa_circ_0012265	Forward	5'-GGTTCCTAAGCCTGTACCACC -3'	qRT-PCR
	Reverse	5'-GCTGGTGCCAAGAATCTCCT-3'	
mDCUN1D4	Forward	5'-TGCAGGCAGTGGACAAGAAA-3'	qRT-PCR RT-PCR
	Reverse	5'-GGATCTTGCTGACCACGACA-3'	
HuR	Forward	5'-ATGTTCTCTCGGTTTGGGCG-3'	qRT-PCR
	Reverse	5'-GTTCTGGTTGGGGTTGGCTG-3'	
18S	Forward	5’ -CGAACGTCTGCCCTATCAACTT-3’	qRT-PCR
	Reverse	5’ -ACCCGTGGTCACCATGGTA-3’	
U6	Forward	5’ -CTCGCTTCGGCAGCACA-3’	qRT-PCR
	Reverse	5’ -AACGCTTCACGAATTTGCG-3’	
GAPDH	Forward	5’ -AGAAGGCTGGGGCTCATTTG-3’	qRT-PCR RT-PCR
	Reverse	5’ -AGGGGCCATCCACAGTCTTC-3’	
DHX9	Forward	5’ -TGCCTCCAAGAAAGTCCA-3’	qRT-PCR
	Reverse	5’ -TCCGCTTCCATTGTCGTAT-3’	
EIF4A3	Forward	5’ -GGGGCATCTACGCTTACGG-3’	qRT-PCR
	Reverse	5’ -GCGATGACATCTCTCCCTTTGA-3’	
QKI	Forward	5’ -CAAACGGAACTCCTCACCC-3’	qRT-PCR
	Reverse	5’ -GCCACCGCACCTAATACAC-3’	
ADAR1	Forward	5’ -CGAGAA TCCCAAACAAGGAA-3’	qRT-PCR
	Reverse	5’ -CTGGATTCCACAGGGATTGT-3’	
TXNIP	Forward	5’ -TGTGTGAAGTTACTCGTGTCAAA-3’	qRT-PCR
	Reverse	5’ -GCAGGTACTCCGAAGTCTGT-3’	
CYP2J2	Forward	5’ -TCCATCCTCGGACTCTCCTAC-3’	qRT-PCR
	Reverse	5’ -GTCACCAAGCTCCAAGCTAAAA-3’	
GPC3	Forward	5’ -CAGTAAGGACTGTGGCCGAAT-3’	qRT-PCR
	Reverse	5’ -AGCAGTACGTTCTCCATGTCAT-3’	
pre-TXNIP	Forward	5’ -AGAAGTTGACTTAGACGGATTGCT-3’	qRT-PCR

	Reverse	5' -ACAGACTATTTCAGTTCAGGTAAGA-3'	
TXNIP-1	Forward	5' -ACGCTTCTTCTGGAAGACCA-3'	qRT-PCR
	Reverse	5' -AGGGGTATTGACATCCACCA-3'	
TXNIP-2	Forward	5' -GCAAGCCTAATGGCTACTCG-3'	qRT-PCR
	Reverse	5' -AGGGGTATTGACATCCACCA-3'	
3' UTR (TXNIP)	Forward	5' -GAGTGTGGGTCCACCTTAGC-3'	qRT-PCR
	Reverse	5' -AGGGGTATTGACATCCACCA-3'	
BEX2	Forward	5' -AAAGAGGAACGAGCGTTAAACA(-3'	qRT-PCR
	Reverse	5' -TCACTAACATTCAAAGGTAGGGC-3'	
DDX6	Forward	5' -ATGGGTCTGTCCAGTCAAAATG-3'	qRT-PCR
	Reverse	5' -GGTGGTCATACTCTGTGCTTG-3'	
FBXW2	Forward	5' -TTCTTTCTCTGACGGACTTGC-3'	qRT-PCR
	Reverse	5' -GCTTGAGGAGAGTCTCTAGGTT-3'	
FXR1	Forward	5' -GAGAGAAGATTTAATGGGCCTGG-3'	qRT-PCR
	Reverse	5' -GCTCAATGGCGGTAACTCCA-3'	
HK2	Forward	5' -GAGCCACCACTCACCCTACT-3'	qRT-PCR
	Reverse	5' -CCAGGCATTCGGCAATGTG-3'	
IFI6	Forward	5' -GGTCTGCGATCCTGAATGGG-3'	qRT-PCR
	Reverse	5' -TCACTATCGAGATACTTGTGGGT-3'	
LOX	Forward	5' -CGGCGGAGGAAAACTGTCT-3'	qRT-PCR
	Reverse	5' -TCGGCTGGGTAAGAAATCTGA-3'	
MGAT5	Forward	5' -CTTCACTCCGTGGAAGTTGTC-3'	qRT-PCR
	Reverse	5' -TGGATGGTAAAGTGCAGAAGC-3'	
PCDH9	Forward	5' -CTGCTCTGATTGCCTGTTTAAGG-3'	qRT-PCR
	Reverse	5' -ACCAGTCTGTAGACAAGGCTG-3'	
PLIN2	Forward	5' -ATGGCATCCGTTGCAGTTGAT-3'	qRT-PCR
	Reverse	5' -GGACATGAGGTCATACGTGGAG-3'	
PML	Forward	5' -CGCCCTGGATAACGTCTTTTT-3'	qRT-PCR
	Reverse	5' -CTCGCACTCAAAGCACCAGA-3'	
SMAD2	Forward	5' -CGTCCATCTTGCCATTCACG-3'	qRT-PCR
	Reverse	5' -CTCAAGCTCATCTAATCGTCCTG-3'	
STXBP4	Forward	5' -CCTTGGCCTGAAGGTACTAGG-3'	qRT-PCR
	Reverse	5' -AGCAGATTCTAACCTCAACTTGG-3'	
ZNF680	Forward	5' -TGGGTGCTCCAGCCTTACT-3'	qRT-PCR
	Reverse	5' -GCAGGCCAGTTAAAAACATTGC-3'	
UQCRB	Forward	5' -GGTAAGCAGGCCGTTTCAG-3'	qRT-PCR
	Reverse	5' -AGGTCCAGTGCCCTCTTAATG-3'	
SMC2	Forward	5' -ACAACACCAGAGTACAGGATCT-3'	qRT-PCR
	Reverse	5' -CGGCCCTGCATGATGAGAA-3'	
DCUN1D4-intron2	Forward	5' -CAGATGCAGGGTGTACAAGG-3'	qRT-PCR
	Reverse	5' -TCAGTAAACAGAGGGGCAACT-3'	

DCUN1D4-intron6	Forward	5’ - CTTCGTGCCCCTCAGTAGTT-3’	qRT-PCR
	Reverse	5’ - CATACCATTGCACTCCAGCC-3’	

Supplementary Table S2 Oligonucleotide sets used for constructs, guide DNA, and probe

Oligo Set	Sequences
pcDNA3.1(+)-circDCUN1D4 (positive)	5’-tactaatgacttttttttttatacttcagATTTTCAGCTGAACTCTCAT-3’(sense)
	5’-gcctaattcttttccttgcttcttacGTTTTCTGGTTCAACACCAA-3’ (antisense)
sh-circDCUN1D4-1	5'-CACCGCAGAAAACATTTTCAGCTGAATTCAAGAGATTCAGCTGAAAATGTTTTCTGTTTTTTG-3' (sense)
	5'-GATCCAAAAAACAGAAAACATTTTCAGCTGAATCTCTTGAATTCAGCTGAAAATGTTTTCTGC-3' (antisense)
sh-circDCUN1D4-2	5'-CACCGCAAACATTTTCAGCTGAACTCAAGAGAAGUUCAGCUGAAAAUGTTTTCTTTTTTG-3' (sense)
	5'-GATCCAAAAAACAAACATTTTCAGCTGAACTCTCTTGAAAGTTCAGCTGAAAATGTTTTC-3' (antisense)
sh-circDCUN1D4-3	5'-CACCGCCCAGAAAACATTTTCAGCTTCAAGAGAAGCTGAAAATGTTTCTGGTTTTTTG-3' (sense)
	5'-GATCCAAAAAACCCAGAAAACATTTTCAGCTCTCTTGAAAGCUGAAAATGTTTCTGGC-3' (antisense)
sh-scb	5'-CACCGCTTCTCCGAACGTGTCACGTTTCAAGAGAACGTGACACGTTCGGAGAATTTTTTG-3' (sense)
	5'-GATCCAAAAAACTTCTCCGAACGTGTCACGTTTCAAGAGAACGTGACACGTTCGGAGAAC-3' (antisense)
sh-HuR	5’-CACCGCCCATCACAGTGAAGTTTGCATTCAAGAGATGCAAACTTCACTGTGATGGGTTTTTTG-3’ (sense)
	5’-GATCCAAAAAACCCATCACAGTGAAGTTTGCATCTCTTGAATGCAAACTTCACTGTGATGGGC-3’ (antisense);
psiCHECK2-HuR-Rluc	5'-TCGAGTGGATACCGTTCGTTGTTAACCGTTGATAGCCTTGGTTTTTAAGCCGTATATGGCTGTAAATAATTGC-3' (sense)
	5'-GGCCGCAATTATTTACAGCCATATACGGCTTAAAAACCAAGGCTATCAACGGTTAACAACGAACGGTATCCAC-3' (antisense)
pcDNA3.1(+)-Flag-HuR	5'-CCGGAATTCATGTCTAATGGTTATGAAGACC-3' (sense);
	5'-CCGCTCGAGTTATTTGTGGGACTTGTTGGTT-3' (antisense)

pcDNA3.1(+)-Flag-HuR(ΔRRM3)	5'-CCGGAATTCATGTCTAATGGTTATGAAGACC-3' (sense);<br>5'-CCGCTCGAGCCAGCCGGAGGAGGCGTTTCCT-3' (antisense)
pcDNA3.1(+)-Flag- HuR (RRM2 + Hinge)	5'-CCGGAATTCATCAAAGACGCCAACTTGTACA-3' (sense);<br>5'-CCGCTCGAGCCAGCCGGAGGAGGCGTTTCCT-3' (antisense)
pcDNA3.1(+)-Flag- HuR (Hinge)	5'-CCGGAATTCAACAAAAACGTGGCACTCCTCT-3' (sense);<br>5'-CCGCTCGAGCCAGCCGGAGGAGGCGTTTCCT-3' (antisense)
psiCHECK2-TXNIP-WT-Rluc	GCATGTGGAAGAAAAGAAGCAGCTTTACCTACTTGTTTCTTTTTGTCTCTCTTC<br>CTGGACACTCACTTTTTCAGAGACTCAACAGTCTCTGCAATGGAGTGTGGGTCC<br>ACCTTAGCCTCTGACTTCCTAATGTAGGAGGTGGTCAGCAGGCAATCTCCTGGG<br>CCTTAAAGGATGCGGACTCATCCTCAGCCAGCGCCCATGTTGTGATACAGGGGT<br>GTTTGTTGGATGGGTTTAAAAATAACTAGAAAAACTCAGGCCCATCCATTTTCT<br>CAGATCTCCTTGAAAATTGAGGCCTTTTCGATAGTTTCGGGTCAGGTAAAAATG<br>GCCTCCTGGCGTAAGCTTTTCAAGGTTTTTTGGAGGCTTTTTGTAAATTGTGAT<br>AGGAACTTTGGACCTTGAACTTACGTATCATGTGGAGAAGAGCCAATTTAACAA<br>ACTAGGAAGATGAAAAGGGAAATTGTGGCCAAAACTTTGGGAAAAGGAGGTTCT<br>TAAAATCAGTGTTTCCCCTTTGTGCACTTGTAGAAAAAAAAGAAAAACCTTCTA<br>GAGCTGATTTGATGGACAATGGAGAGAGCTTTCCCTGTGATTATAAAAAAGGAA<br>GCTAGCTGCTCTACGGTCATCTTTGCTTAGAGTATACTTTAACCTGGCTTTTAA<br>AGCAGTAGTAACTGCCCCACCAAAGGTCTTAAAAGCCATTTTTGGAGCCTATTG<br>CACTGTGTTCTCCTACTGCAAATATTTTCATATGGGAGGATGGTTTTCTCTTCA<br>TGTAAGTCCTTGGAATTGATTCTAAGGTGATGTTCTTAGCACTTTAATTCCTGT<br>CAAATTTTTTGTTCTCCCCTTCTGCCATCTTAAATGTAAGCTGAAACTGGTCTA<br>CTGTGTCTCTAGGGTTAAGCCAAAAGACAAAAAAAATTTTACTACTTTTGAGAT<br>TGCCCCAATGTACAGAATTATATAATTCTAACGCTTAAATCATGTGAAAGGGTT<br>GCTGCTGTCAGCCTTGCCCACTGTGACTTCAAACCCAAGGAGGAACTCTTGATC<br>AAGATGCCCAACCCTGTGATCAGAACCTCCAAATACTGCCATGAGAAACTAGAG<br>GGCAGGTCTTCATAAAAGCCCTTTGAACCCCCTTCCTGCCCTGTGTTAGGAGAT<br>AGGGATATTGGCCCCTCACTGCAGCTGCCAGCACTTGGTCAGTCACTCTCAGCC<br>ATAGCACTTTGTTCACTGTCCTGTGTCAGAGCACTGAGCTCCACCCTTTTCTGA<br>GAGTTATTACAGCCAGAAAGTGTGGGCTGAAGATGGTTGGTTTCATGTTTTTGT<br>ATTATGTATCTTTTTGTATGGTAAAGACTATATTTTGTACTTAACCAGATATAT<br>TTTTACCCCAGATGGGGATATTCTTTGTAAAAAATGAAAATAAAGTTTTTTTAA<br>TGGAAAAAAAAA

psiCHECK2-TXNIP-Mut-Rluc	GCATGTGGAAGAAAAGAAGCAGCTTTACCTACTTGTTTCTTTTTGTCTCTCTTC<br>CTGGACACTCACTTTTTCAGAGACTCAACAGTCTCTGCAATGGAGTGTGGGTCC<br>ACCTTAGCCTCTGACTTCCTAATGTAGGAGGTGGTCAGCAGGCAATCTCCTGGG<br>CCTTAAAGGATGCGGACTCATCCTCAGCCAGCGCCCATGTTGTGATACAGGGGT<br>GTTTGTTGGATGGGTTTAAAAATAACTAGAAAAACTCAGGCCCATCCATTTTCT<br>CAGATCTCCTTGAAAATTGAGGCCTTTTCGATAGTTTCGGGTCAGGTAAAAATG<br>GCCTCCTGGCGTAAGCTTTTCAAGGTTTTTTGGAGGCTTTTTGTAAATTGTGAT<br>AGGAACTTTGGACCTTGAACTTACGTATCATGTGGAGAAGAGCCAATTTAACAA<br>ACTAGGAAGATGAAAAGGGAAATTGTGGCCAAAACTTTGGGAAAAGGAGGTTCT<br>TAAAATCAGTGTTTCCCCTTTGTGCACTTGTAGAAAAAAAAGAAAAACCTTCTA<br>GAGCTGATTTGATGGACAATGGAGAGAGCTTTCCCTGTGATTATAAAAAAGGAA<br>GCTAGCTGCTCTACGGTCATCTTTGCTTAGAGTATACTTTAACCTGGCTTTTAA<br>AGCAGTAGTAACTGCCCCACCAAAGGTCTTAAAAGCCATTTTTGGAGCCTATTG<br>CACTGTGTTCTCCTACTGCAAATATTTTCATATGGGAGGATGGTTTTCTCTTCA<br>TGTAAGTCCTTGGAATTGATTCTAAGGTGATGTTCTTAGCACTTTAATTCCTGT<br>CAAATTTTTTGTTCTCCCCTTCTGCCATCTTAAATGTAAGCTGAAACTGGTCTA<br>CTGTGTCTCTAGGGTTAAGCCAAAAGACAAAAAAAATTTTACTACTTTTGAGAT<br>TGCCCCAATGTACAGAATTATATAATTCTAACGCTTAAATCATGTGAAAGGGTT<br>GCTGCTGTCAGCCTTGCCCACTGTGACTTCAAACCCAAGGAGGAACTCTTGATC<br>AAGATGCCCAACCCTGTGATCAGAACCTCCAAATACTGCCATGAGAAACTAGAG<br>AAACCTTTTCATAAAAGCCCTTTGAACCCCCTTCCTGCCCTGTGTTAGGAGAT<br>AGGGATATTGGCCCCTCACTGCAGCTGCCAGCACTTGGTCAGTCACTCTCAGCC<br>ATAGCACTTTGTTCACTGTCCTGTGTCAGAGCACTGAGCTCCACCCTTTTCTGA<br>GAGTTATTACAGCCAGAAAGTGTGGGCTGAAGATGGTTGGTTTCATGTTTTTGT<br>ATTATGTATCTTTTTGTATGGTAAAGACTATATTTTGTACTTAACCAGATATAT<br>TTTTACCCCAGATGGGGATATTCTTTGTAAAAAATGAAAATAAAGTTTTTTTAA<br>TGGAAAAAAAAA
psiCHECK2-AluJo-WT-Rluc	GCTTACATTTTTATTGCCTAATATATATGCAAACATACTTTACTTCAAGTTGAG<br>GCTGCGCGTAGTGGCCCACACCTTTAATCCCAACATTTTGGGAGGCTGAGGCAG<br>GAGGATCACTTGAACCCAGGAGTTCAAGACCAGCCTGGGAAACATAGAAAGACC<br>CTGTCTCTACAAGAAAAAAAAAAAAGAAAAATTAGCTGTGCATGATGGTGCACAC

psiCHECK2-AluJo-Mut-Rluc	GCTTACATTTTTATTGCCTAATATATATGCAAACATACTTTACTTCAAGTTGAG<br>GCTGCGCGTAGTGGCCCACACCTTTAAAGGGTTGATTTTGGGAGGCTGAGGCAG<br>GAGGATCACTTGAACCCAGGAGTTCAAGACCAGCCTGGGAAACATAGAAAGACC<br>CTGTCTCTACAAGAAAAAAAAAAAGAAAAATTAGCTGTGCATGATGGTGCACAC
psiCHECK2-AluSc-WT-Rluc	GTAGCTGGGACTACAGGTGTGCGCCACCATGCTTGGCTAATGTTTTTTGTATTT<br>TAGTAGAGATGGGATTTCACCATGTTGGCCAGATGTCTCTATCTCCTGACCTTG<br>TGATCTGCCCGGCTCAGCCTCCCAAAGTGCTGGGATTATAGGCGTGAGCCACAG<br>CACCCGGCCATGATTGCTTTTTATAATGTAAAAGCCCTAGGTATTTAT

psiCHECK2-AluSc-WT-Rluc	GTAGCTGGGACTACAGGTGTGCGCCACCATGCTTGGCTAATGTTTTTTGTATTTTAGTAGAGATGGGATTTCACCATGTTGGCCAGATGTCTCTATCTCCTGACCTTGTGATCTGCCCGGCTCAGCCAGGGTTTGTGCTGGGATTATAGGCGTGAGCCACAGCACCCGGCCATGATTGCTTTTTATAATGTAAAAGCCCTAGGTATTTAT
CircDCUN1D4 junction probe (northern blot)	5'-GTTGAGAGATGAGAGTTCAGCTGAAAATGTTTTCTGGTTCAACACCAATGTCTTC- DIG-3'
CircDCUN1D4 junction probe (pull down)	5'-GTTGAGAGATGAGAGTTCAGCTGAAAATGTTTTCTGGTTCAACACCAATGTCTTC-3'
CircDCUN1D4 Scramble probe	5'-GTGTAACACGTCTATACGCCCA-3'
CircDCUN1D4 FISH Probe	5'-GAAAACAUUUUCAGCUGAATT-3' (sense)<br>5'-UUCAGCUGAAAAUGUUUUCUG-3' (antisense)
CircDCUN1D4 ISH Probe	5'-GTTGAGAGATGAGAGTTCAGCTGAAAATGTTTTCTGGTTCAACACCAATGT CTTC-3'

Supplementary Table S3 Target sequences of siRNA

Oligo Set	Target sequences
si-ADAR1-1	GCAGAGTCAGCATATATGA
si-ADAR1-2	GGCCCGAGATATAAATGCT
si-ADAR1-3	GGCTCTCCGTGTCTTGATT
si-DHX9-1	CGAACACCATTGCATGAAA
si-DHX9-2	GGACTAGTAGCAACATTGA
si-DHX9-3	GCATGGACCTCAAGAATGA
si-QKI-1	GAAGCTGGTTTAATCTATA
si-QKI-2	GACCTATTGTTCAGTTACA
si-QKI-3	GAAACCGGATGTAAAATCA
si-EIF4A3-1	CGAGCAATCAAGCAGATCA
si-EIF4A3-2	GCTGGATTACGGACAGCAT
si-EIF4A3-3	CTCTCGGTGACTACATGAA
si-TXNIP-1	CAACATCCTTCGAGTTGAA
si-TXNIP-2	ACACGCTTCTTCTGGAAGA
si-TXNIP-3	GTCTGTCTCTGCTCGAATT

Supplementary Table S4 candidate targets of circDCUN1D4

BEX2	CYP2J2	DDX6
FBXW2	FXR1	GPC3
HK2	IFI6	LOX
MGAT5	PCDH9	PLIN2
PML	SMAD2	SMC2
STXBP4	TXNIP	UQCRB
ZNF680		

Table S5 Pathological and clinical data of lung cancer cases in tissue microarray

Variables	Cases(n) (total n=92)	circDCUN1D4 Low(n)	circDCUN1D4 High(n)	P value
Age(yes)				
≤60	38	14	24	0.731
>60	54	18	36	
Gender				
Male	51	22	29	0.062
Female	41	10	31	
Tumor size				
≤5cm	69	23	46	0.618
>5cm	23	9	14	
Lymph node invasion				
Present	41	15	26	0.008
Absent	51	17	34	
TNM stage				
I	7	3	4	0.045
II-Ⅲ	85	29	56	
EGFR mutation				
Present	22	9	13	0.495
Absent	70	23	47	

Table S6 Univariate analysis and Multivariable analysis

Parameters	Univariate analysis			Multivariable analysis		
	HR	95%CI	P value	HR	95%CI	P value
Age(>60)	2.701	1.466-4.975	0.001	2.383	1.266-4.483	0.007
Gender(Male)	1.501	0.60-1.865	0.869	—	—	—
Lymph node metastasis(Positive)	3.297	1.857-5.855	<0.0001	2.002	1.034-3.875	0.039
Pathological grading(G2-G4)	4.709	3.144-6.366	<0.0001	3.805	2.540-5.091	0.002
Tumor diameter(>5cm)	4.150	2.185-7.884	0.031	1.676	0.756-3.716	0.204
EGFR mutation(positive)	4.563	2.427-8.577	0.215	1.405	0.636-3.107	0.401
circDCUN1D4 level(low)	2.250	1.285-3.940	0.005	1.593	1.205-2.922	0.033
